# A Chemist’s Guide to Solid-State Qubits: Diamond Color Centers as Embedded Vacancy-Centered Molecules

**DOI:** 10.1021/acscentsci.6c00548

**Published:** 2026-07-27

**Authors:** John Mark P. Martirez

**Affiliations:** Applied Materials and Sustainability Sciences, Princeton Plasma Physics Laboratory, Princeton, New Jersey 08540-6655, United States

## Abstract

Diamond point defects or color centers are at the crux of solid-state quantum technology applications, from computing to sensing. Modeling molecular qubits has advanced by leaps and bounds mainly due to the advanced quantum mechanical (QM) tools in the arsenal of quantum chemists for modeling (isolated) molecular systems, which should be easily translatable for analogous systems in the solid state. Here, we simulate a series of diamond defects: a carbon vacancy (V_C_) and second row substitutions that are adjacent to a V_C_ or *D*_C_V_C_, with *D* = B, N, and O, to evaluate their magnetic and optical properties. We applied molecular orbital principles, an embedded cluster method, and a multiconfigurational QM theory to computationally characterize the above-mentioned defects for a multitude of spin, charge, and excited states. Our computational approach delivered high-quality QM insights that compare well with experiments. Furthermore, with this framework, we confirm the spin-triplets B_C_V_C_^–^ and O_C_V_C_^0^ to be analogous to N_C_V_C_^–^, with calculated bright transitions that respectively correspond to UV (3.80 eV) and blue (2.65 eV) light absorption and emission lifetimes (τ) of 2.3 and 5.6 ns, which are higher in energy and more short-lived (and thus brighter) than that for N_C_V_C_^–^: green (2.26 eV), τ = 9.5 ns.

## Graphical abstract

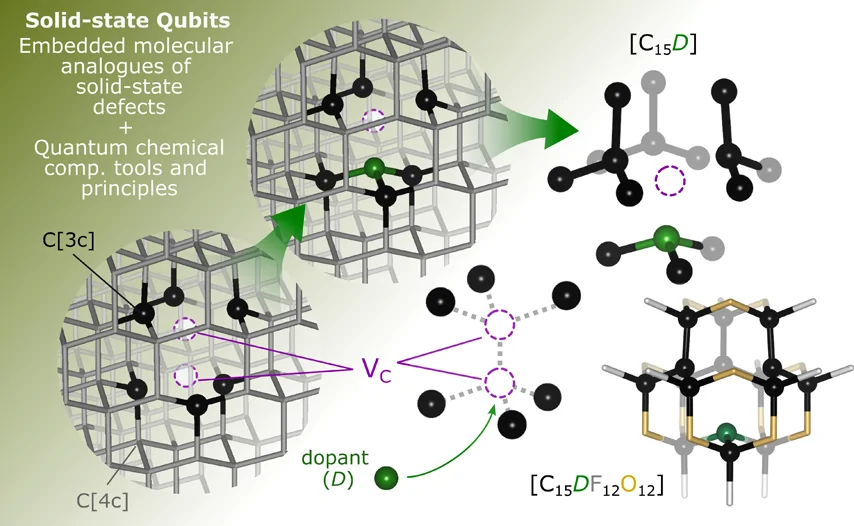

## Introduction

Solid-state quantum defects have emerged as promising basic units of information in devices for quantum technology, for example, as “quantum bits” or “qubits” in quantum computing, communication, network, and sensing.^1–4^ These technologies aim to take advantage of quantum phenomena, specifically quantum superposition and entanglement. Qubits can be manipulated by a static or oscillating magnetic or electric field, e.g., visible light and microwaves. A unit of information can be written, transmitted, or read through the qubits’ (quantum) responses to perturbations.^1–4^

Qubit hosts therefore ideally should be diamagnetic (i.e., “nonmagnetic”) and a wide-band gap insulator to ensure that the host does not strongly interfere with the qubit and magnetic and optical perturbations.^3,5^ One such example is diamond, which is among most the studied materials for solid-state quantum applications. In addition to possessing the properties described above (magnetic susceptibility^6^ of −4.5 × 10^–7^ emu/g and band gap^7^ of 5.5 eV), diamond has high thermal conductivity and a relatively high dielectric constant^8^ (*ε*_*r*_ = 5.35); it can be made with high purity and crystallinity and has great potential for precise engineering to contain desired defects.^9,10^ Diamond has been shown to host a variety of magnetically and optically active point defects, e.g., neutral and charged vacancies, substitutions, interstitials, and some combinations, yielding different optical absorption profiles and colorations.^11–14^ Because of their varied coloration, such defects are referred to as color centers.

Among the notable examples of color centers are carbon vacancies (V_C_s), interstitial atoms (e.g., H and Si (H_*i*_ and Si_*i*_)), substitutional atoms (e.g., N and B (N_C_ and B_C_)), and defects resulting from their pairing, e.g., N_C_V_C_ and V_C_Si_*i*_V_C_, commonly referred to as NV and SiV centers, respectively.^13^ N_C_, N_C_V_C_, and V_C_ defects are naturally occurring in nitrogen-containing diamond of any origin^13^ and can be synthesized by N ion implantation into diamond.^13^ V_C_s can aggregate and manifest various spectroscopic signatures. Prominent examples are the so-called ND1 and GR1 centers, which are ascribed to a negatively charged and a neutral isolated single V_C_, respectively.^13,15^ The negatively charged N_C_V_C_ (N_C_V_C_^–^) is among the most interrogated defects in diamond and regarded as a promising qubit. N_C_V_C_^–^ is generated by irradiating diamond samples containing N_C_V_C_ and donor defects, such as N_C_ and phosphorus substitutions (P_C_), with electrons, e.g., a 10^22^ m^–2^ 2 MeV electron beam.^16^ N_C_V_C_^–^ has a spin-triplet ground state and a higher energy spin-singlet (a magnetically inactive shelving state, accessible via nonradiative intersystem crossing from the excited triplet) that luminesce in the IR (1.19 eV). The spin-triplet has a strong visible light emission (red, 1.95 eV) after green light absorption (e.g., 2.33 eV).^16,17^ N_C_V_C_^0^ and N_C_V_C_^+^ may coexist with N_C_V_C_^–^,^18,19^ where the former has a spin-doublet ground state, whereas the latter has a spin-singlet ground state. The ability to delineate contributions from the different charge states of qubits (that may be advertently or inadvertently present) in the optical and magnetic response of the sample can enable more refined quality assessment and characterization, leading to a more targeted and judicious qubit manipulation and read-out. Indeed, precise characterization of the magnetic and optical properties of defects is required for the development of application-specific qubits with desired properties. For example, computing applications require qubits with long spin coherence lifetimes at or near room temperature and resilience to environmental perturbations to reduce noise. Establishing rules for the discovery of new dopant-based quantum defects (including codoping) and alternative hosts is still emerging and concurrently being explored with clever ways to manipulate and probe qubits.^1,2,5,12,20^ Quantum mechanics (QM) simulations with accurate electronic structure theory to understand and characterize the magnetic and optical properties of existing and candidate qubits is valuable in exploring such new opportunities.

Qubits have a multiconfigurational electronic character, i.e., strongly correlated, which motivates the importance of using accurate multireference QM theories to predict their optical and magnetic properties. With qubits, one would need to determine (at the minimum) the ground-state spin, existence of other (less-stable but accessible) spin multiplicities (via intersystem crossing, ISC), absorption and emission frequencies (i.e., zero-phonon line or ZPL and phonon sidebands in absorption and photoluminescence spectra), excited-state lifetimes, ground- and excited-state zero-field splitting (ZFS) parameters between the spin sublevels (*m*_s_, e.g., *m*_s_ = 0 and ±1 for a spin-triplet), and nuclear spins and their coherence lifetimes. A practical way to simulate qubits (molecular or solid-state) quantum mechanically would be within density functional theory (DFT).^21–24^ However, DFT is a ground-state theory and its most widely used implementation, Kohn–Sham DFT, uses only one electronic configuration (single reference) to represent the electronic structure of a chemical system. The accuracy of DFT also rests on the approximation of the electron exchange and correlation (XC) potentials (via an XC density functional). Furthermore, for spin-polarized systems, spin symmetry is typically broken in DFT (separate orbitals for the spin “up” and “down” electrons). Consequently, although spin-unrestricted Kohn–Sham wavefunctions remain eigenfunctions of *Ŝ*_*z*_, they are no longer guaranteed to be eigenfunctions of *Ŝ*^2^, possibly leading to spin contaminated solutions (spin symmetry breaking is often required to obtain the variationally lowest energy solutions for single reference methods). As the field of quantum materials moves further into the wider composition and phase space to identify novel quantum defects and hosts, the use of rigorous electronic structure theories coupled with sound modeling frameworks would greatly complement and augment experiments, making experimental campaigns into the unknown composition and phase spaces with greater confidence.

Simulation of molecular qubits has progressed by leaps and bounds mainly due to the advanced QM tools in the arsenal of quantum chemists for modeling (isolated) molecular systems.^25,26^ These methods, so-called post-Hartree–Fock or correlated wavefunction (CW) theories, utilize the exact Fock exchange and incorporates correlation via expansion of the wavefunction in the electron configuration space, i.e., configuration interaction (CI). For excited states and generally systems with strong (static) correlation, multiconfigurational CW methods are paramount, e.g., the complete active space self-consistent field method (CASSCF)^27,28^ with second-order perturbation (PT2) correction (with two variants: CASPT2 ^29–31^ and the *n*-electron valence state PT2 or NEVPT2)^32^ and multireference singles and doubles CI (MRSDCI).^33^ These methods, however, scale ∼*N* ^4^ to *N* ^6^, where *N* represents the system size (e.g., number of orbitals or electrons), making them applicable only for small to medium sized chemical systems. Furthermore, one typically uses QM codes that use periodic boundary conditions (PBCs) to simulate crystalline materials. However, most of such codes that utilize PBCs, primarily due to computational cost, implement only DFT or higher-level but single-reference methods, e.g., random-phase approximation (RPA),^34–37^ *GW* and *GW* with the Bethe–Salpeter equation,^38–45^ Møller–Plesset perturbation theory,^46–50^ and coupled cluster.^50–52^ Although recently, multideterminant quantum Monte Carlo calculations were performed using periodic cells for the N_C_V_C_^–^ defect in diamond, they were not without associated quantitative limitations.^53^ CW-based simulations of defects require a reduction in model size, e.g., as a molecular species or atomic cluster, assuming the defect electronic states are localized. Simulation of defects in diamonds via the molecular fragment approach is not without precedent. Among the earliest simulations of such kind are those by Coulson and co-workers in 1957 and 1971 (V_C_ with 0, +, and – charges)^54,55^ and by Surratt and Goddard in 1977 (V_C_^0^).^56^ In the former, the authors approximated a V_C_ as isolated orbitals of the four C atoms surrounding the vacancy in an “infinite matrix”,^54,55^ whereas in the latter the authors used the smallest possible C cluster capped with H atoms (C_4_H_12_).^56^ In the former, the authors used a CI method using minimal Slater and Clementi basis orbitals, yielding somewhat qualitatively consistent predictions with experiments, albeit with quantitatively mixed results. In the latter, the authors employed generalized valence bond CI method with classical polarization correction to calculate a rudimentary approximation of the first excited state for the spin-singlet and spin-triplet states of V_C_^0^ in an infinite solid. The authors found a nontrivial polarization correction of ∼5 eV to the calculated excitation energies, which proved to be necessary to achieve good agreement with experiments.^56^ There are now a plethora of cluster-type simulations of diamond defects, e.g., the N_C_V_C_^–^,^57–61^ in the literature. For example, Benedek et al. recently showed that a H-capped 145-C atom cluster (C_145_H_148_N^–^) is needed to achieve convergence in excitation energies within CASSCF-NEVPT2.^60^ That said, embedded cluster approaches with CW methods have now also been widely used in the literature to characterize diamond-based defects that explicitly account for the effect of the extended diamond environment within a lower-level theory without the need for large explicitly correlated atomic clusters.^57,62–67^

Here, we use a recently benchmarked best-of-both-worlds approach,^67^ where we use hypothetical V_C_-centered molecules (capped C atom clusters) to represent the defects and use our recently developed capped density functional embedding theory (capped-DFET)^68,69^ to derive the influence of a periodic diamond lattice environment to said hypothetical molecules. In doing so, we were able to perform nonperiodic accurate CW simulations to characterize the electronic and magnetic properties of defects while also accounting for bulk crystal effects in a cost-effective manner.^67^ In this work, with capped-DEFT, we were able to explore many dopant (*D*)–vacancy pairs (*D*_C_V_C_), with *D* = B, N, and O, and V_C_ (i.e., *D* = C) in diamond for different charge and spin states, as well as calculate a multitude of excited states. We also characterized the electronic structure of the defects in the frameworks of molecular orbitals and group theories. Both N_C_V_C_ and V_C_ have been thoroughly studied in the experimental and computational literature, which can thus serve as benchmarks for the simulations. B_C_ and O_C_ paired with V_C_(s), on the other hand, are being examined for their viability as alternatives to N_C_V_C_ as qubits.^70–73^ Their implementation could thus benefit from high-quality ab initio simulation data to be fully realized.

## Results and Discussion

### Overview of the Modeling Framework

*D*_C_V_C_ pairs consist of three coordinatively unsaturated C atoms and a heteroatom (the dopant) surrounding a V_C_, where the latter tunes the former’s electronic structure. To provide a simple picture on the nature of the electronic structure of a *D*_C_V_C_, we consider the introduction of *D* into one of the two adjacent V_C_s (Figure 1A); note, however, that this need not be the actual mechanism by which these defects form. If *D* is almost similar in size to C (second period main group elements), it will simply fill one of the V_C_ sites. The three 3-fold coordinated C atoms (C­[3c]­s) around the V_C_, the three C atoms nearest and bound to *D* (C­[n*D*]­s), and *D* itself yield orbitals distinct from the host and impart the defect with its characteristic electronic structure (vide infra).

**Figure 1:**
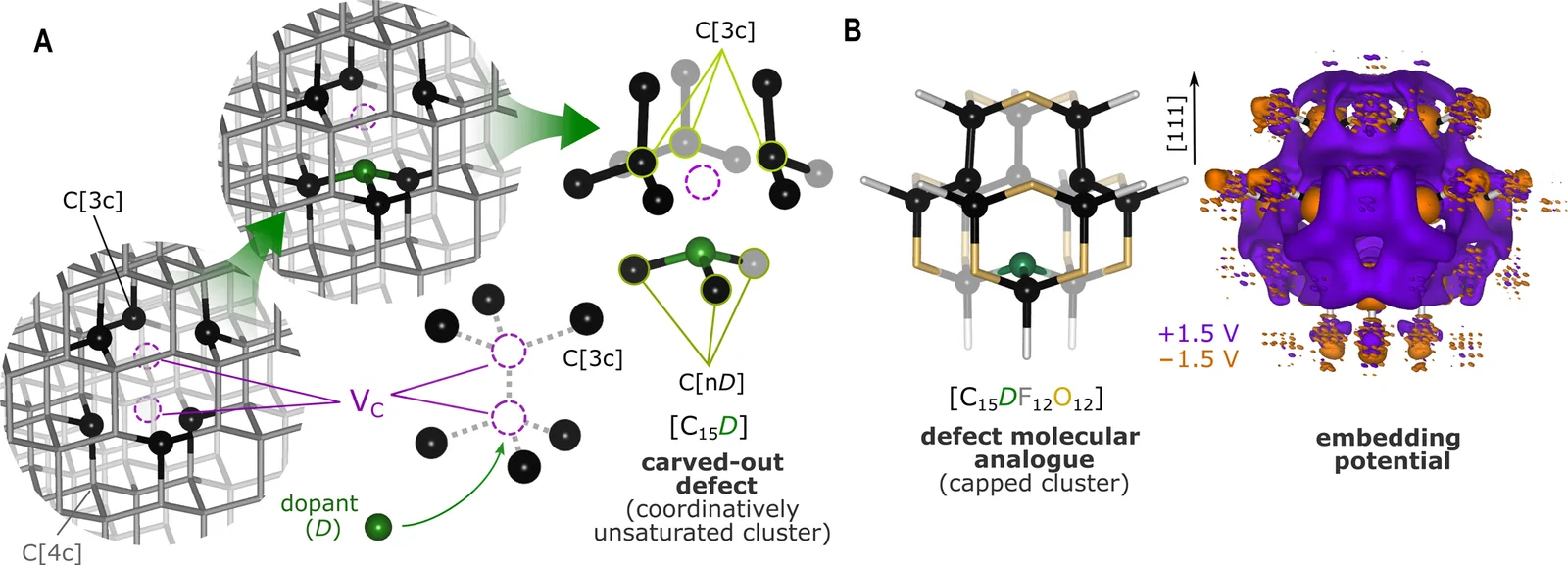
Atomic structure of *D*_C_V_C_ defects. (A) Left: a diamond lattice (4-fold coordinated C atoms, C­[4c]) containing two adjacent C vacancies (V_C_), where each of the six surrounding C atoms is only 3-fold coordinated (C­[3c]). Center: upon implantation of a period 2 atom (*D* = B, C, N, and O) at a V_C_, the implanted atom forms bonds with three of the six C­[3c] atoms, i.e., the C atoms nearest to *D* (C­[n*D*]). Right: a *D*_C_V_C_ defect can thus be defined by the three C­[3c]­s, *D*, nine C atoms nearest to the C­[3c]­s, and three C­[n*D*]­s, where together they yield C_15_ *D*. (B) Left: C_15_ *D* fragment capped with F (monovalent capping) and O (divalent capping) generates a V_C_-centered defect molecular analogue: C_15_ *D*F_12_O_12_. Right: isosurface of an optimized embedding potential evaluated from capped-DFET that captures the interaction of the defect with the diamond lattice (not accounted for by the capping elements) and thus serves to replicate the defect’s and diamond host’s mutual dielectric responses. Purple: repulsive; orange: attractive potential.

Therefore, to simulate *D*_C_V_C_ (*D* = B, N, and O) and V_C_ (i.e., *D* = C) defects, we first build periodic defective C_126_ *D* diamond structures for multiple charge states. We structurally optimized these models within DFT using the VASP^74,75^ code using a deorbitalized meta generalized gradient approximation XC functional, namely, r^2^-SCAN-L.^76,77^ DFT-r^2^-SCAN-L reproduces the experimental cubic lattice constant of pure diamond within 0.0006 Å (Table S1). Using these optimized structures, we re-evaluated the ground-state electron densities and total energies using DFT with a more accurate hybrid HSE06 XC functional^78,79^ (see Methods in the Supporting Information, Periodic DFT calculations). We carved out clusters from the C_126_ *D* supercells (Figure S1) that include *D*, the three C­[3c]­s around V_C_, the nine nearest carbon neighbors of the three C­[3c]­s, and the three C­[n*D*]­s, which yield C_15_ *D* atomic clusters (Figure 1A). The C atoms at the edges of the C_15_ *D* clusters are coordinatively unsaturated. We therefore capped these atoms with F (for monovalent capping, i.e., terminal atoms) or O (for divalent capping, i.e., bridging atoms) to yield C_15_ *D*F_12_O_12_ clusters—hypothetical V_C_-centered molecules (Figure 1B). We then derived the influence of the periodic diamond lattice on these molecules, i.e., we calculated optimized effective embedding potentials (*V*_emb_), via capped-DFET within the DFT-HSE06 level of theory (Figure 1B); see Methods, Embedding and fragmentation scheme and Capped-DFET formalism, and Figures S2 (fragmentation and capping scheme) and S3 (*V*_emb_ isosurface plots). Using these nonperiodic embedded molecules, we performed state-averaged complete active space self-consistent field (SA-CASSCF)^27,28^ calculations followed by the *n*-electron valence state second-order perturbation theory (NEVPT2)^32^ within Molpro^80,81^ to calculate defect ground- and excited-state energies and many-body wavefunctions using the Gaussian-type orbital (GTO) basis set (see Methods, Excited state calculations). Henceforward, we refer to these simulations as embedded SA-CASSCF (emb-SA-CASSCF) and embedded NEVPT2 (emb-NEVPT2). In ref 67, we extensively benchmarked this modeling scheme for N_C_V_C_^–^ with respect to the (1) underlying DFT approximation in the *V*_emb_ optimization, (2) capped cluster and periodic supercell sizes, (3) GTO basis set size, and (4) CASSCF active space size and character. From emb-SA-CASSCF and emb-NEVPT2 simulations of the hypothetical molecules, we calculated vertical excitation energies (VEEs), excited-state transition dipole moments (μ_t_), oscillator strengths (*f*), and natural lifetimes (τ) (see Methods, Excited state properties).

### Structural Symmetry and the Electronic Ground State of the Defects from Molecular Orbital Theory

One may understand the electronic structure of the defect site using molecular orbital (MO) theory. For illustrative purposes, we consider the hybridized 2*sp* ^3^ orbital for C because it serves as an intuitive basis of the covalent bond network in diamond. The four valence orbitals of *D* = B, C, N, and O (one 2*s* and three 2*p*) mix with three 2*sp* ^3^ orbitals from the three C­[n*D*]­s (Figure 1, left panel). Specifically, the *D* atom forms three bonding (σ) and three antibonding (σ*) orbitals with C­[n*D*]­s at the substitution site. The three nonbonding 2*sp* ^3^ orbitals of C­[3c]­s and a 2*p* orbital of *D* (or another 2*sp* ^3^ for *D* = C = C­[3c]) form the basis of the four V_C_-centered nonbonding defect orbitals. Because the total number of orbitals is conserved, these orbital interactions yield a total of 10 defect orbitals. Figure 2 illustrates these orbital interactions (there are 10) for *D* = B, C, and jointly, N and O, which shows orbital occupation for the negatively charged defect for *D* = B, C, and N and the neutral defect for *D* = O (N_C_V_C_^–^ and O_C_V_C_^0^ are isoelectronic). Orbitals are not unique and can be changed via unitary transformations while not affecting the total energy and electron density. The defect orbitals illustrated in Figure 2 are idealized symmetry adapted linear combination (SALC) orbitals, which aide in the discussion of the symmetry of the electronic states. We label these idealized SALC orbitals in Figure 2 (and similarly, the calculated orbitals, vide infra) by their irreducible representations (irreps) following the Mulliken notation^82^ as prescribed by the defect’s (determined) point group symmetry and expected/calculated orbital degeneracies.

**Figure 2:**
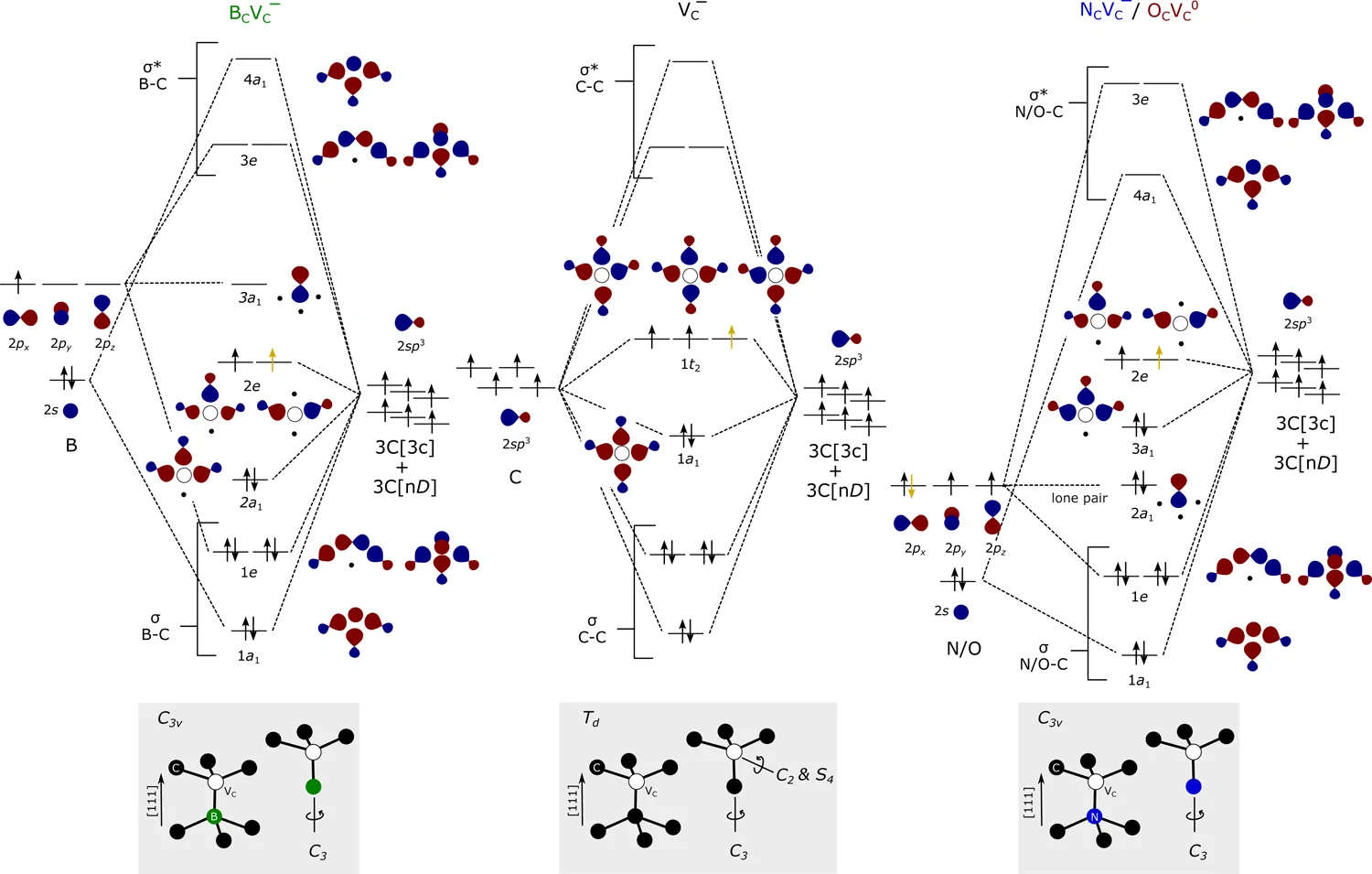
Molecular orbital (MO) diagrams of the B_C_V_C_, V_C_, N_C_V_C_, and O_C_V_C_ color centers in diamond. Top row: MO diagrams of the spin-triplet B_C_V_C_^–^, N_C_V_C_^–^, and O_C_V_C_^0^ and spin-quartet V_C_^–^, which retain ideal defect symmetry. N_C_V_C_^–^ and O_C_V_C_^0^ are isoelectronic. The orbital interactions are portrayed in the basis of the atomic-like valence 2*s* and three 2*p* orbitals of *D* ≠ C (four 2*sp* ^3^ orbitals in *D* = C) and a total of six 2*sp* ^3^ orbitals from the three C­[3c] and three C­[n*D*] atoms at the vacancy and substitution sites. Bottom row: schematics of the local defect structure showing notable symmetry operations, i.e., examples of axes of rotation. The MO diagrams illustrate the molecular (not atomic) nature of the electronic structure of the color centers in diamond, where the orbitals of the C atoms at the defect sites are paramount. The up and down arrows refer to electrons with up and down spins. The gold up or down arrow marks the excess electron for the anions (there is no excess electron in O_C_V_C_^0^). The defect orbitals are labeled according to the defect’s irreducible representation in their symmetry group following the Mulliken notation.

The bottom insets in Figure 2 show the defects’ basic structural units and symmetries (gray box). The highest symmetry a *D*_C_V_C_-type defect (*D* ≠ C) can adopt is C_3*v*_ (Schoenflies notation) with a characteristic 3-fold axis of rotation (*C*_3_). V_C_ (*D* = C) without structural distortions (Jahn–Teller distortions, vide infra), inherits the local tetrahedral symmetry (*T*_*d*_) of pure diamond, with characteristic *C*_3_ and 2-fold (*C*_2_) axes of rotation and 4-fold (*S*_4_) improper axes of rotation (rotation plus reflection perpendicular to the rotational axis). In all four defects in Figure 2, the three lowest energy defect valence orbitals are the three fully occupied bonding σ orbitals of the *D*–C bond (1*a*_1_ and doubly degenerate 1*e* for *C*_3*v*_), with their corresponding empty antibonding σ* orbitals (4*a*_1_ and 3*e*) as the three highest energy defect valence orbitals. We do not label these six orbitals as such in *D* = C because all four C­[3c]­s are identical and there are in fact a total of (4 C­[3c] × 6 orbitals = ) 24 C–C σ and σ* orbitals. All 24 orbital linear combinations should then be labeled using irreps within the *T*_*d*_ symmetry (which would be cumbersome). The defects differ in character in their four nonbonding frontier orbitals—the orbitals that give rise to the defects’ magnetic and optical properties relevant to qubit operation, which we discuss further below.

The nonbonding frontier valence orbitals of B_C_V_C_^–^ (Figure 2, left panel) are, namely, a mirror and *C*_3_ rotationally symmetric *a*_1_ orbital (2*a*_1_) with all three C­[3c] 2*sp* ^3^ orbitals in-phase, two (degenerate) 2*e* orbitals where two or three C­[3c] 2*sp* ^3^ orbitals are out-of-phase, and another *a*_1_ orbital (3*a*_1_) that is derived from the empty nonbonding B 2*p* orbital. These orbitals are analogous to the four occupied valence orbitals of NH_3_,^83^ but here only 2*a*_1_ is doubly occupied, whereas the 2*e* orbitals are singly occupied and 3*a*_1_ is empty. The 2*e* orbitals thus constitute the highest occupied molecular orbitals (HOMOs), whereas the 3*a*_1_ is the lowest unoccupied MO (LUMO). When accounting for all the orbitals derived from the dopant and vacancy, the full 10 orbital set would be analogous to an ethane molecule with a missing C atom or C_1_H_6_. In such a comparison, the (1*a*_1_, 1*e*, 3*a*_1_, 3*e*, 4*a*_1_) orbital set constitutes and is isolobal with the CH_3_^+^ fragment MOs. The (2*a*_1_, 2e) set then is isolobal with the three linear combinations of the three 1s orbitals of H_3_^–^ surrounding the missing C atom. For V_C_^–^ (Figure 2, center panel), *T*_*d*_ symmetry prescribes a low energy totally symmetric orbital (1*a*_1_) and three degenerate (*S*_4_ improper rotationally antisymmetric) orbitals (1*t*_2_). The 1*a*_1_ orbital has all four C­[3c] 2*sp* ^3^ orbitals in-phase, whereas the 1*t*_2_ orbitals have two pairs of C­[3c] 2*sp* ^3^ out-of-phase. These orbitals are analogous to the four occupied valence orbitals of CH_4_; however, here only 1*a*_1_ is doubly occupied, whereas all three 1*t*_2_ orbitals are singly occupied. The orbital occupation schemes (simply following Hund’s rule) give rise to a spin-triplet (*s* = 1 or 2*s* + 1 = 3) B_C_V_C_^–^ and a spin-quartet (*s* = 3/2 or 2*s* + 1 = 4) V_C_^–^. N_C_V_C_^–^ and O_C_V_C_^0^ are isoelectronic (Figure 2, right panel) and, like B_C_V_C_^–^, belong to the *C*_3*v*_ point group symmetry. They also have two *a*_1_ and two *e* frontier valence orbitals. However, unlike in B_C_V_C_^–^, both *a*_1_ orbitals are fully occupied, where the lower energy orbital is associated with the dopant’s lone pair electrons (now 2*a*_1_), whereas the higher energy *a*_1_ orbital is the in-phase combination of the three nonbonding C­[3c] 2*sp* ^3^ (now 3*a*_1_). The two 2*e* orbitals constitute the two HOMOs. The two 2*e* orbitals remain singly occupied and thus yield a ground-state spin-triplet (*s* = 1).

Removal of an electron from the above-mentioned defects results in B_C_V_C_^0^, V_C_^0^, N_C_V_C_^0^, and O_C_V_C_^+^ defects and leads to uneven occupancy of the degenerate *e* or *t*_2_ HOMOs (Figure 2). B_C_V_C_^0^, N_C_V_C_^0^, and O_C_V_C_^+^ are thus spin-doublets (*s* = 1/2; 2*s* + 1 = 2), whereas V_C_^0^ is an open-shell spin-singlet (*s* = 0; 2*s* + 1 = 1, vide infra). We found that the uneven occupancies of degenerate orbitals result in structural symmetry breaking, i.e., Jahn–Teller distortion^83^ to eliminate or reduce HOMO degeneracy leading to energy lowering and lower symmetry (vide infra). Finally, removal of one electron from B_C_V_C_^0^, N_C_V_C_^0^, and O_C_V_C_^+^ yields B_C_V_C_^+^, N_C_V_C_^+^, and O_C_V_C_^2+^ and completely empties their 2*e* orbitals with a doubly occupied *a*_1_ (2*a*_1_ for B_C_V_C_^0^ and 3*a*_1_ for N_C_V_C_^+^ and O_C_V_C_^2+^) becoming the HOMO (Figure 2). With all the degenerate states becoming completely empty, we found that the *C*_3*v*_ symmetry is restored (vide infra). B_C_V_C_^+^, N_C_V_C_^+^, and O_C_V_C_^2+^ now have no unpaired electrons and are thus spin-singlets (*s* = 0; 2*s* + 1 = 1) and magnetically inactive in their ground state.

### Structural Symmetry and the Electronic Ground State of the Defects from Simulations

As stated above, we constructed the V_C_-centered molecular analogues (C_15_ *D*F_12_O_12_, Figure 1) for multiple charge states from the spatially periodic supercell simulations of the defects (Table S2—optimized lattice constants without imposing symmetry), using their respective ground state spin multiplicity (Note S1 and Table S3—energy differences between different spin multiplicities; Figure S4—DFT-HSE06 spin density isosurface plots). We confirm that the local defect structures optimized from DFT- r^2^-SCAN-L and DFT-HSE06 (using lattice constants from DFT- r^2^-SCAN-L optimization) can be very similar (Methods, Periodic DFT calculations, and Table S4).

We determined the symmetry of the C_15_ *D*F_12_O_12_ molecular analogues via the continuous symmetry measure method^84^ (Note S2 and Tables S5 and S6). The top row of Figure 3 illustrates the local defect structure (showing only the three C­[3c]­s and *D* atom) and shows the evolution of the defects’ symmetry as their charge changes. The figure shows the nearest C­[3c]–C­[3c] and C­[3c]–*D* distances to illustrate local symmetry. The middle row of Figure 3, on the other hand, shows the defects’ frontier orbitals and (one of) their ground-state (primary) electron configurations (note that the majority of these defects requires more than a single determinant, i.e., electronic configuration, to properly describe their electronic structures). We found that the calculated emb-SA-CASSCF active space natural orbitals (resulting from the diagonalization of the state-averaged density matrix, Figures S5–S8) can be easily mapped visually to the SALC orbitals illustrated in Figure 2 and that they exhibit symmetries consistent with the symmetries of the atomic structures even without imposing constraints. These orbitals were labeled according to their (approximate) irreps (given the defect’s point group symmetry), which we determined via visual inspection and consulting character tables (Table S7), and are reflected in Figure 3. We confirm from our periodic DFT-HSE06 and capped cluster emb-NEVPT2 simulations that the favored spin multiplicities of the defects in their different charge states (Table S3 and Note S1) are what they are predicted to be from MO theory (vide supra), illustrating the utility of the MO framework. MO theory also proved to be valuable in determining the active space character in emb-SA-CASSCF simulations (Methods, Excited state calculations). Furthermore, as stated above, the uneven occupancies of the degenerate *e* or *t*_2_ orbitals in B_C_V_C_^0^, V_C_^0^, N_C_V_C_^0^, and O_C_V_C_^+^ lead to Jahn–Teller distortions, thereby reducing orbital degeneracy. This is indeed the case for these defects; for example, compare the nearest C­[3c]–C­[3c] and C­[3c]–*D* distances for the different charge states of the same dopant in the top row of Figure 3. In B_C_V_C_^0^, N_C_V_C_^0^, and O_C_V_C_^+^, one of the three nearest C­[3c]–C­[3c] and C­[3c]–*D* distances is longer and shorter, respectively, than the other two, which removes five out of the six symmetry operations associated with the *C*_3*v*_ point group (including *C*_3_), retaining only one reflection symmetry (σ_*h*_) that cuts through the elongated nearest C­[3c]–C­[3c] distance. B_C_V_C_^0^, N_C_V_C_^0^, and O_C_V_C_^+^ thus belong to the *C*_*s*_ point group (Table S5), where *a*_1_ becomes a reflection symmetric *a*′, whereas the two *e* orbitals become nondegenerate reflection symmetric and antisymmetric *a*′ and *a*″ orbitals (Table S7 and Figure 3, middle row, for panels B_C_V_C_, N_C_V_C_, and O_C_V_C_). As for V_C_^0^, we found that two of the closest C­[3c]–C­[3c] distances are longer than the other four (Figure 3, top row), reducing the number of symmetry operations from 24 (*T*_*d*_) to 8 (Table S6). Notably, V_C_^0^ retains two *S*_4_ axes and two dihedral mirror planes (σ_*d*_), where some are illustrated in the top row of Figure 3 in the V_C_ panel. V_C_^0^ thus belongs to the *D*_2*d*_ point group, where *a*_1_ remains *a*_1_ but the *t*_2_ orbitals become two degenerate *C*_2_-antisymmetric *e* orbitals and a nondegenerate *b*_2_ orbital that is antisymmetric to both *S*_4_ and *C*_2_′ (Table S7 and Figure 3, middle row for V_C_). Note that *S*_4_ and *C*_2_ (not *C*_2_′) share the same axis (Figure 3, top row, V_C_ panel). We calculated the energy of the corresponding symmetric structures, i.e., structures without Jahn–Teller distortions, for B_C_V_C_^0^, V_C_^0^, N_C_V_C_^0^, and O_C_V_C_^+^ by imposing symmetry on the atomic (and by extension electronic) structures. We define the Jahn–Teller distortion energy (Δ*E*_JT_) as the energy difference between the Jahn–Teller distorted and their corresponding symmetric structures. Δ*E*_JT_ measures the stability gained upon symmetry breaking. Figure S9 shows the optimized C­[3c]–C­[3c] and C­[3c]–*D* distances with symmetry imposed and the −Δ*E*_JT_s. Figure S9 shows positive −Δ*E*_JT_s that are below and above the thermal energy at room temperature (298 K) (*k*_B_ *T* ∼ 0.025 eV) that ranges from 0.01 to 0.56 eV. −Δ*E*_JT_ ∼ 0.25 to 0.56 eV or ∼10*k*_B_ *T* to 22*k*_B_ *T* (N_C_V_C_^0^ and V_C_^0^) indicates a frozen defect structure (static Jahn–Teller distortion) at room and colder temperatures, whereas 0.01 to 0.14 eV or ∼0.4*k*_B_ *T* to 6*k*_B_ *T* (B_C_V_C_^0^ and O_C_V_C_^+^) will lead to zero-point-energy and thermally induced structural fluctuations. Note that there are three degenerate Jahn–Teller distorted structures with *C*_*s*_ and *D*_2*d*_ symmetry for the *C*_3*v*_ and *T*_*d*_ point groups, respectively. This is because one of the three symmetry-equivalent σ planes or *S*_4_ axes is preserved (while the other two are broken) for a given *C*_*s*_ or *D*_2*d*_ distortion, respectively (Tables S5 and S6). Temperature-induced structural fluctuations when −Δ*E*_JT_ ≲ 0.1 eV (dynamic Jahn–Teller distortion)^91^ can give rise to either the *C*_3*v*_ or *T*_*d*_ average structure due to the equal probability of forming the three degenerate distorted structures with a small barrier for transformation. For O_C_V_C_^+^, since −Δ*E*_JT_ is still ∼6*k*_B_ *T*, the structure still spends a majority of the time distorted with $3/\left[3+e^{\Delta E_{\mathrm{JT}}/k_{B}T}\right]$ ∼ 1.00 normalized probability at *k*_B_ *T* = 0.025 eV (room temperature), where the factor 3 is due to the three degenerate Jahn–Teller distorted configurations. For B_C_V_C_^0^, because −Δ*E*_JT_ < *k*_B_ *T* (although still positive), the defect will be represented by both *C*_*s*_ and *C*_3*v*_ structures with 0.84 and 0.16 normalized probabilities at *k*_B_ *T* = 0.025 eV, respectively. Here, for B_C_V_C_^0^, V_C_^0^, N_C_V_C_^0^, and O_C_V_C_^+^, we present only the result for the Jahn–Teller distorted structures, although for B_C_V_C_^0^, the small −Δ*E*_JT_ warrants further investigation of the *C*_3*v*_ structure in the future, due to its finite contribution (16%).

**Figure 3:**
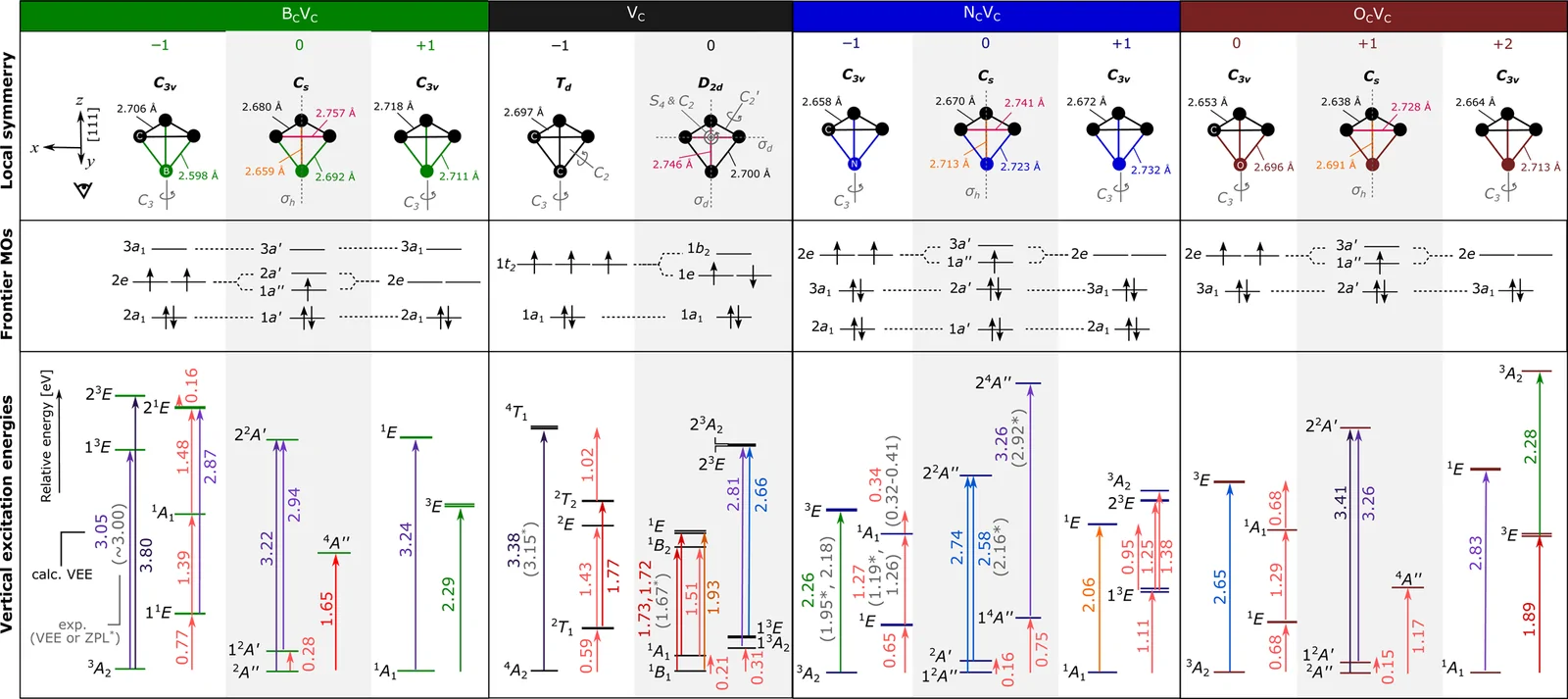
Structure, ground-state electronic configurations, and excited-state diagrams of *D*_C_V_C_ color centers with *D* = B, C, N, and O for different charge (and spin) states. Top row: local defect structures showing the three C­[3c]­s and *D* (as labeled). The nearest C­[3c]–C­[3c] and C­[3c]–*D* distances (annotated) of the same length (within ±0.001 Å) have the same color. The nearest C­[3c]–C­[3c] and C­[3c]–*D* distances illustrate the local symmetry of the defects: *C*_3*v*_, *C*_*s*_, *T*_*d*_, or *D*_2*d*_. Some of the notable symmetry operations (rotation: *C*_3_ and *C*_2_; improper rotation: *S*_4_; horizontal and dihedral reflection: σ_*h*_ and σ_*d*_) are illustrated. *x* = [101̅], *y* = [1̅21̅], and *z* = [111]. Middle row: frontier MOs and the primary ground-state electronic configurations. The MOs are labeled by their irreps using Mulliken notation (see also Figure 2 for the MO diagrams and Figures S5–S8 for the calculated emb-SA-CASSCF natural orbitals corresponding to these orbitals). Bottom row: Jablonski energy diagrams. Ground and excited electronic states (Mulliken labels) and VEEs from emb-NEVPT2 with emb-SA-CASSCF. The VEEs are marked with vertical arrows (to scale) and annotated. The arrows are colored according to the VEEs’ corresponding color in the visible spectrum (1.7 to 3.3 eV; red to violet), infrared (<1.7 eV; pink), and UV (>3.3 eV; deep violet). Experimental (exp.) ZPL (marked with *) or VEE values in gray: B_C_V_C_, ref 70; V_C_, refs 85 and 86; N_C_V_C_, refs 16, 17, and 87–90 . Zero-phonon line (ZPL) accounts for phonon and then electronic relaxation following vertical excitation. VEE corresponds to the excitation from the structural ground state to an electronically excited state that may be in a higher vibronic state. VEE is thus ≥ZPL.

In B_C_V_C_^+^, N_C_V_C_^+^, and O_C_V_C_^2+^, the equivalences of all three C­[3c]–C­[3c] and C­[3c]–*D* distances are restored, and *C*_3*v*_ symmetry re-emerges (Figure 3, top row). The degeneracy between *a*′ and *a*″ orbitals recovers back to *e*, and the other two *a*′ orbitals become *a*_1_ orbitals (Figure 3, middle row). The re-emergence of the high-symmetry defect structures is due to the complete emptying of the degenerate *e* orbitals.

In the next section, we present the lowest energy electronic excitation profiles of the defects. We also calculate electronic transitions for the higher energy spin multiplicity, namely, for B_C_V_C_, N_C_V_C_, and O_C_V_C_: a singlet (*s* = 0) for a ground-state triplet, a quartet (*s* = 3/2) for a ground-state doublet, and a triplet (*s* = 1) for a ground-state singlet (Table S3). For V_C_, we calculate a doublet (*s* = 1/2) for a ground-state quartet and a triplet (*s* = 1) for a ground-state singlet (Table S3). Figure S10 shows the corresponding electronic configurations for these higher energy spin states.

### Defects’ Excited-State Characters and Energies

Using the symmetry of the defects as a guide in determining possible state degeneracies, we calculate (and label accordingly) the lowest-lying excited states using emb-SA-CASSCF. We then calculate VEEs from emb-NEVPT2. The zero-phonon lines (ZPLs) of the color centers are typically reported due to their sharp spectral feature. ZPLs account for phonon and then electronic relaxation following an electronic vertical excitation. On the other hand, VEEs (fixed nuclei approximation) correspond to the excitation from the structural ground state to an electronically excited state that may be in a higher vibronic state: VEE is thus ≥ZPL. Experimental VEEs are identified as one of the phonon sideband peaks in the absorption spectra. Typically, the difference between VEE and ZPL for diamond defects is equivalent to the energy of the second or third vibrational quantum number of the excited electronic state where the ZPL can be lower by ∼0.2–0.3 eV (vide infra). VEEs thus serve as a guide on the proper excitation wavelength of the color center, while ZPL is the fluorescence signal to measure.

From the lowest energy electronic configurations for each spin multiplicity (Figure 3, middle row, and Figure S10), one may infer that the lowest energy defect-centered valence excitations for B_C_V_C_ are primarily from 2*a*_1_ to 2*e* and from 2*e* to 3*a*_1_ for the cation and anion, respectively, and from 1*a*′ to (1*a*″, 2*a*′) and from (1*a*″, 2*a*′) to 3*a*′ for the neutral one (Figure S5—emb-SA-CASSCF active space natural orbitals of B_C_V_C_^–,0,+^). For V_C_, defect-centered excitations are primarily from 1*a*_1_ to 1*t*_2_ for the negative defect and from 1*a*_1_ to 1*e* and from 1*e* to 1*b*_2_ for the neutral defect (Figure S6—emb-SA-CASSCF active space natural orbitals of V_C_^–,0^). Finally, for N_C_V_C_ and O_C_V_C_, these excitations primarily originate from 3*a*_1_ to 2*e* for N_C_V_C_^–,+^ and O_C_V_C_^0,2+^ and from 2*a*′ to (3*a*′, 1*a*″) for N_C_V_C_^0^ and O_C_V_C_^+^. We found for N_C_V_C_ that the valence electron correlation and low energy excitations have some contributions from 2*a*_1_ (Figure S7—emb-SA-CASSCF active space natural orbitals of N_C_V_C_^–,0,+^), but not for O_C_V_C_ (Figure S8—emb-SA-CASSCF active space natural orbitals of O_C_V_C_^0,+,2+^). The lack of the static correlation contributions of the O_C_ lone pair in the ground and excited states is due to O’s higher nuclear charge (than N), which yields a much lower energy 2*a*_1_ (lone pair) orbital. The 2*a*_1_ orbital is thus not included in the active space for any charge state of O_C_V_C_. Tables S8 and S9 summarize the emb-SA-CASSCF contributions of the dominant electronic configurations for each state to the many-body wave function via the configuration interaction (CI) coefficients (*c*_*i*_) in the basis of the state-averaged natural orbitals (Figures S5–S8). As expected, for many of the states (especially for degenerate and near degenerate states), more than one electronic configuration dominates the wave function expansion, far from a single-particle picture (that Kohn–Sham DFT provides).

Through the SA-CASSCF many-body wave functions, we also characterized the excitations via the charge density change (showing where the excited electron and hole reside), transition dipole moments (showing absorption strength and polarization of light that will be maximally adsorbed), oscillator strengths (transition probability with an ideal value of 1 for single-electron excitations, which is a measure of brightness), and natural emission lifetime. We labeled the electronic states according to their irreps as dictated by the capped cluster’s symmetry (*C*_3*v*_, *C*_*s*_, *T*_*d*_, or *D*_2*d*_). The irreps are determined by the state’s degeneracy (single *A*, *B*; double *E,* triple *T*) and the dominant electron configurations as measured by the configurations’ CI coefficients *c*_*i*_ (Tables S8 and S9). For *C*_3*v*_ and *C*_*s*_ point groups, the electron configurations were used along with the frontier orbital irreps (*a*[_1,2_^′,″^], *b*[_1,2_], *e*, *t*[_1,2_]) and symmetry product table (Table S10). For *T*_*d*_ and *D*_2*d*_ point groups, we consult the table of allowed states for V_C_^–,0^ with *T*_*d*_ symmetry determined by Coulson and Kearsley (Table S11)^55^ along with the calculated dominant electron configurations (Tables S8). For V_C_^0^, which reduces to *D*_2*d*_, we use irrep mapping between *T*_*d*_ and *D*_2*d*_ (Table S7). Note that we did not impose any of the identified symmetry in the electronic structure calculations; therefore, state degeneracies and orbital symmetries naturally arise from the cluster’s structural symmetry (see Methods, Excited state calculations). We followed emb-SA-CASSCF with state-specific emb-NEVPT2 to calculate vertical excitation energies (VEEs). The bottom row of Figure 3 shows the Jablonski diagrams for the low-lying valence excited states (annotated with VEEs) for all the defects of varying charge states. Below we discuss these valence excited states and their energy profiles per dopant type.

### B_C_V_C_’s Optical Transitions

B_C_V_C_^–^ has a ^3^ *A*_2_ ground state with the lowest triplet excited state being both doubly degenerate: 1^3^ *E* and 2^3^ *E*, which absorb violet to UV and are 0.75 eV from each other (Figure 3, bottom row, first panel). 1^3^ *E* is a combination of excitations from 2*e* to 3*a*_1_ and 2*a*_1_ to 2*e* orbitals, with the former having higher *c*_*i*_s (Table S8). Excitation into 3*a*_1_ is possible because B has an empty electron acceptor 2*p* orbital (3*a*_1_). The predicted first excitation energy, ^3^ *A*_2_ → 1^3^ *E* (3.05 eV), matches well with the 3 eV experimental minimum excitation energy for an optical pump spin polarization observed in B_C_V_C_^–^.^70^ 2^3^ *E* is more predominantly excitations from 2*a*_1_ to 2*e* with a much higher VEE of 3.80 eV (but still below the band gap of diamond). Figure S11 shows that the charge density redistribution for ^3^ *A*_2_ to 1^3^ *E* and 2^3^ *E*. Consistent with the *c*_*i*_s, the density difference plot shows that the two 1^3^ *E* deplete electrons from C­[3c]­s, which contribute most to 2*e*, and add electron density to B. 2^3^ *E*, on the other hand, involves repolarization of the electron density at the V_C_, where a C­[3c] gains electrons (two C­[3c]­s lose) in one and the same C­[3c] loses electrons (two C­[3c]­s gain) in the other. Therefore, 2^3^ *E* (not 1^3^ *E*) is analogous to the lowest excited ^3^ *E* of N_C_V_C_^–^ (vide infra), contrary to the assumption in a recent experiment.^70^ On the other hand, the higher energy spin-singlet has a doubly degenerate ground state (1^1^ *E*) resulting from the spin-pairing of the 2*e* electrons. The first excited state (1^1^ *A*_1_), where all electrons are paired for each occupied orbital, includes a contribution from the double excitation from 2*a*_1_ to 2*e* (Table S8). 1^1^ *A*_1_ may luminesce infrared and is below 1^3^ *E.* The second excited singlet is doubly degenerate (2^1^ *E*) and primarily involves an excitation from 2*e* to 3*a*_1_. 2^1^ *E* is just below 2^3^ *E* (∼0.2 eV) and above 1^3^ *E.* 2^1^ *E* can thus serve as a shelving state for 2^3^ *E* and luminesces IR or UV when relaxing to 1^1^ *A*_1_ or 1^1^ *E*, respectively (Figure 3, bottom row, first panel).

B_C_V_C_^0^ has a ^2^ *A*″ ground state (Figure 3, bottom row, second panel) with a first excited state (1^2^ *A*′) that is very close in energy (∼0.3 eV). ^2^ *A*″ and 1^2^ *A*′ result from the splitting of a ^2^ *E* state due to symmetry lowering (*C*_3*v*_ → *C*_*s*_), i.e., Jahn–Teller distortion (vide supra). 1^2^ *A*′ is simply the promotion of the unpaired electron from 1*a*″ to 2*a*′ (Table S8). The second excited state (2^2^ *A*′) is the excitation from 1*a*″ to 3*a*′, where 3*a*′ is the B 2*p* lone pair. Therefore, 2^2^ *A*′ depletes electron density from C­[3c]­s, which it adds to B (Figure S11). The higher energy quartet state (1^4^ *A*″) is in between ^2^ *A*″ and 2^2^ *A*′ and is characterized by single-electron occupation of 1*a*′, 1*a*″, and 2*a*′. The first and second quartet excited states are nearly degenerate and are two linear combinations of what would be ^4^ *A*′ and 2^4^ *A*″ states (not shown in Figure 3). The VEEs are 3.8–4.1 eV and involve excitations from 1*a*″ and 2*a*′ to 3*a*′ (Table S8). Because of the large energy misalignment between the doublet and quartet states, intersystem crossing (ISC) between these spin multiplicities is very unlikely.

B_C_V_C_^+^ restores *C*_3*v*_ symmetry and has a ground state ^1^ *A*_1_, where 2*e* is completely empty (now the LUMO) and the HOMO becomes the doubly occupied 2*a*_1_. Excitations (involving both one and two electrons, Table S8) from 2*a*_1_ to 2*e* yield a doubly degenerate first excited state (^1^ *E*) absorbing UV (Figure 3, bottom row, third panel). The ^1^ *E* state is a repolarization of the V_C_ electron density and does not involve B (Figure S11). The lowest energy triplet (^3^ *E*) has one electron each in 2*a*_1_ and 2*e*, a spin-flip state of ^1^ *E*. Excitation of the 2*a*_1_ electron to 2*e* yields ^3^ *A*_2_, where there is one electron on each of the 2*e* orbitals and has a VEE of 2.67 eV, way above ^1^ *E* (not shown in Figure 3).

### V_C_’s Optical Transitions

The V_C_^–^ defect is distinct from the other defects due to its higher symmetry. Its ground state is a spin-quartet ^4^ *A*_2_ (Figure 3, bottom row, fourth panel). The first excited state is a triply degenerate state (^4^ *T*_1_) resulting from the excitation of an 1*a*_1_ electron into a 1*t*_2_ orbital, where one of the three singly occupied 1*t*_2_ orbitals becomes doubly occupied and there are three such ways to fill *t*_2_ (Table S8). This excitation occurs by the absorption of low UV (3.38 eV). V_C_^–^ (also known as the ND1 center) has a measured strong PL with a ZPL of 3.149 eV.^86,92^ The absorption phonon sidebands are ∼3.23, 3.30, and 3.38 eV, suggesting VEEs of 3.2–3.4 eV,^92^ which our calculation reproduces well. The lowest energy spin-doublet is about half an eV higher than the ground state and is triply degenerate (^2^ *T*_1_). It results from the spin-pairing of the two of the three *t*_2_ electrons relative to ^4^ *A*_2_. We calculated two distinct doublet excited states: ^2^ *E* and ^2^ *T*_2_, which are 1.43 (infrared) and 1.77 (red) eV above ^2^ *T*_1_. ^2^ *T*_2_ remains ∼1 eV below the ^4^ *T*_1_.

The V_C_^0^ defect, which has *D*_2*d*_ symmetry, has a nondegenerate ground state (^1^ *B*_1_) but a low energy first excited state (^1^ *A*_1_). ^1^ *B*_1_ and ^1^ *A*_1_ result from the splitting of a ^1^ *E* state due to the Jahn–Teller distortions (Figure 3, bottom row, fifth panel). In ^1^ *B*_1_, each of the 1*e* orbitals is singly occupied of the opposite spin. The dominant configurations of ^1^ *A*_1_ have all electrons paired within an orbital where either the 1*b*_2_ or (to a lesser extent) one of the two 1*e* is fully occupied (Table S8). The next three higher excited states, ^1^ *B*_2_ and ^1^ *E*, result from the splitting of a ^1^ *T*_2_ state. ^1^ *B*_2_ shares similar characters as ^1^ *A*_1_, where either one of the 1*e* orbitals is doubly occupied. ^1^ *E* has 1*e* and 1*b*_2_ singly occupied (two permutations, Table S8). The ^1^ *B*_2_ absorbs red (1.73 eV), while ^1^ *E* absorbs orange (1.93 eV). V_C_^0^ (also known as the GR1 center) has a measured ZPL with two peaks at 1.673 and 1.665 eV.^13^ Emissions ^1^ *B*_2_ → ^1^ *B*_1_ and ^1^ *E* → ^1^ *A*_1_, respectively, after the ^1^ *B*_1_ → ^1^ *E* excitation could yield a doublet-like photoluminescence profile at 1.73 and 1.72 eV, which can potentially explain the experimentally measured doublet ZPL in the PL spectra. We calculated two low-lying spin-triplets: 1^3^ *A*_2_ and 1^3^ *E*, which are only 0.31 and 0.46 eV higher than the ground state ^1^ *B*_1_. They arise from the splitting of 1^3^ *T*_1_ when going from *T*_*d*_ to *D*_2*d*_ symmetry (Tables S7 and S11). Similarly, the higher spin-triplet states are 2^3^ *E* and 2^3^ *A*_2_, which result from the splitting of the 2^3^ *T*_1_ state. 2^3^ *E* and 2^3^ *A*_2_ (2.8 eV from 1^3^ *A*_2_) are nearly degenerate, indicating a weak Jahn–Teller effect. They are way above either of the excited spin-singlets ^1^ *B*_2_ and ^1^ *E*.

### N_C_V_C_’s and O_C_V_C_’s Optical Transitions

Finally, we turn our attention to *D* = N and O. Because N_C_V_C_^–/0/+^ are isoelectronic to O_C_V_C_^0/1+/2+^, they have similar magnetic and excitation profiles (with a few exceptions), and thus, we discuss them jointly. We previously presented a comprehensive benchmarking of our method used herein for N_C_V_C_^–^.^67^ We present some of the results in ref 67 but refined our calculation of ^3^ *A*_2_ → ^1^ *E* (Methods) and report here other excited-state properties not calculated before (vide infra). Likewise, in a separate publication, we performed similar simulations for the neutral and +2-charged O_C_V_C_ and a double-vacancy complex V_C_O_C_V_C_.^93^ There, we show that the experimentally observed O_C_V_C_-derived color center, specifically the ST1 color center,^71^ is the V_C_O_C_V_C_^2+^ defect.^93^ Here, we present results from O_C_V_C_^0/2+^ (now including the +1-charged defect) to compare with the different charge states of N_C_V_C_. N_C_V_C_ presents opportunities to benchmark our predictions (as with V_C_, vide supra), and thus, we mention any experimental data when available.

The dopants appear to simply tune the excited-state energetics, mostly quantitatively, but sometimes, they also tune the character of the state. Of note, because of the much lower energy of the lone pair (2*a*_1_) of O_C_ than that of N_C_, the 2*a*_1_ orbital does not participate in the static correlation of O_C_V_C_’s valence electrons for any charge state. Otherwise, N_C_V_C_ and O_C_V_C_ share the same frontier orbital character (Figures S7 and S8). Both N_C_V_C_^–^ and O_C_V_C_^0^ have a nondegenerate spin-triplet ground state (^3^ *A*_2_) and an accompanying doubly degenerate first excited state (^3^ *E*) (Figure 3, bottom row, sixth and ninth panels). These excitations occur in the visible spectrum, green (2.26 eV) and blue (2.65 eV) in N_C_V_C_^–^ and O_C_V_C_^0^, respectively.^67,93^ Both defects also have a low-lying doubly degenerate spin-singlet (^1^ *E*) that is ∼0.7 eV from the ground state ^3^ *A*_2_. Their excited-state spin-singlets (^1^ *A*_1_) fluoresce in the infrared to their respective lowest energy spin-singlet ^1^ *E*.^67,93^ The ISC between ^3^ *E* and ^1^ *A*_1_ is likely more probable for N_C_V_C_^–^ than for O_C_V_C_^0^ due to the smaller energy difference between the aforementioned states (0.37 vs 0.68 eV). Our calculated VEEs and ISC energy for N_C_V_C_^–^ compare very well (within 0.1 eV) with experimental literature data.^66,67^ O_C_V_C_^0^ appears to be a suitable alternative to N_C_V_C_^–^ but requires a blue laser (instead of green) as an optical pump. We found recently that O_C_V_C_^0^ is unstable relative to the formation of a divacancy complex forming V_C_O_C_V_C_^0^, which is favored by the stability of the two lone pair electrons on O (O, after all, prefers to form only two covalent bonds).^93^

N_C_V_C_^0^ and O_C_V_C_^+^ exhibit Jahn–Teller distortions resulting in the ground state ^2^ *E* splitting into ^2^ *A*″ and ^2^ *A*′ (Figure 3, bottom row, seventh and 10th panels), similar to B_C_V_C_^0^, with a Jahn–Teller splitting of ∼0.2 eV. The second excited state is either ^2^ *A*″ (N_C_V_C_^0^) or ^2^ *A*′ (O_C_V_C_^+^). Excitation to the second excited state correspond to the visible light absorption in the blue (2.74 eV, N_C_V_C_^0^) and near UV (3.41 eV, O_C_V_C_^+^). Calculated emission lifetimes (vide infra) indicate equal rates of de-excitation from the second excited state to the first excited and ground states. Relaxation from the second excited states to the first excited state (assuming no structural relaxation) yields 2.58 and 3.26 eV for N_C_V_C_^0^ and O_C_V_C_^+^, respectively. A ZPL from photoluminescence experiments registers at 2.16 eV for N_C_V_C_^0^,^90^ indicating 0.4 eV in structural relaxation prior to and post electronic relaxation. The lowest energy quartet state ^4^ *A*″ for both defects is 0.75 and 1.2 eV higher in energy than the spin-doublet. The energy to excite ^4^ *A*″ of N_C_V_C_^0^ is 3.3 eV with the promotion of a spin-down 1a′ electron to 2a′ (Table S9). We expect that the excitation of ^4^ *A*″ O_C_V_C_^+^ to require even higher energy because of the much lower 1a′ (we did not explore this further).

Finally, for N_C_V_C_^+^ and O_C_V_C_^2+^, a spin-singlet ground state (^1^ *A*_1_) yields first excited states that absorb 2.06 and 2.83 eV and are characterized to be ^1^ *E* (Figure 3, bottom row, eighth and 11th panels). Because the 2*e* orbitals are now completely empty in ^1^ *A*_1_, excitation to ^1^ *E* involves the transition of a 3*a*_1_ electron to one of the 2*e* orbitals (Table S9). The ^1^ *E* states are higher in energy than the lowest energy spin-triplets that are also doubly degenerate (1^3^ *E*). ^3^ *E* is the spin-flip state of 1^1^ *E*. The spin-triplet N_C_V_C_^+^ and O_C_V_C_^2+^ have an excited state ^3^ *A*_2_. Furthermore, N_C_V_C_^+^ has a lower energy 2^3^ *E* triplet excited state that originates from the 2*a*_1_ to 3*a*_1_ excitation and is nearly degenerate with ^3^ *A*_2_. We found that the 2^3^ *E* state is higher in energy than ^3^ *A*_2_ in O_C_V_C_^2+^ by performing a five-state emb-SA-CASSCF simulation with a larger active space of (8e,9o), where we explicitly include the 1*e* and 2*a*_1_ orbitals and additional correlating virtual orbitals.^93^ We note that when performing emb-NEVPT2, the state-specific treatment of the spin-triplet excited states when including both ^3^ *A*_2_ and 2^3^ *E* together with 1^3^ *E* in state-averaging for O_C_V_C_^2+^ (a five-state emb-SA-CASSCF calculation) appears to result in an unphysically much lower ^3^ *A*_2_ energy compared to the three-state calculation (^3^ *A*_2_ + 1^3^ *E*). Such behavior may mean that the wavefunctions from the five-state SA-CASSCF have become poor references for the lower energy states (SA-CASSCF solves for a compromise set of orbitals for all averaged states). Alternatively, it could be that a proper state mixing via multistate multireference PT2 ^94^ is needed when solving for more states simultaneously in the case of O_C_V_C_^2+^, but such a scheme is currently not implemented with NEVPT2. We therefore do not report the VEEs from the five-state calculation for O_C_V_C_^2+^. Both N_C_V_C_^+^ and O_C_V_C_^2+^, just like B_C_V_C_^+^, appear to be viable spin-singlet qubits with 1^3^ *E* as a metastable spin-triplet shelving state. N_C_V_C_^+^ is typically difficult to observe in NV-containing diamond and was only directly probed recently via positron annihilation spectroscopy with in situ light illumination.^18^

### Transition Dipole Moments (“Brightness” of Excitation)

Although charge density differences (Figures S11–S14) are useful in characterizing an excitation, it does not necessarily show the dipole of the excitation. The transition dipole moment vector (**μ**_**t**_) depends on the spatial overlap of the initial and final state wave functions and corresponds to the direction of polarization of light needed to maximize absorption. The square of its magnitude (|**μ**_**t**_|^2^) is proportional to the absorption strength, which in turn dictates emission intensity (brightness) and lifetime (inverse proportionality) (Methods, Excited state properties). Figure 4 shows the transition dipole moment vectors and magnitude (for multidegenerate excitations, only one is represented) for select excitations for the preferred spin multiplicity for each defect type and charge. Table S12 summarizes all transition dipole moment components for all spin-allowed excitations shown in Figure 3 from the ground state (including excitation from the first excited state for Jahn–Teller-broken ground-state degeneracies).

**Figure 4:**
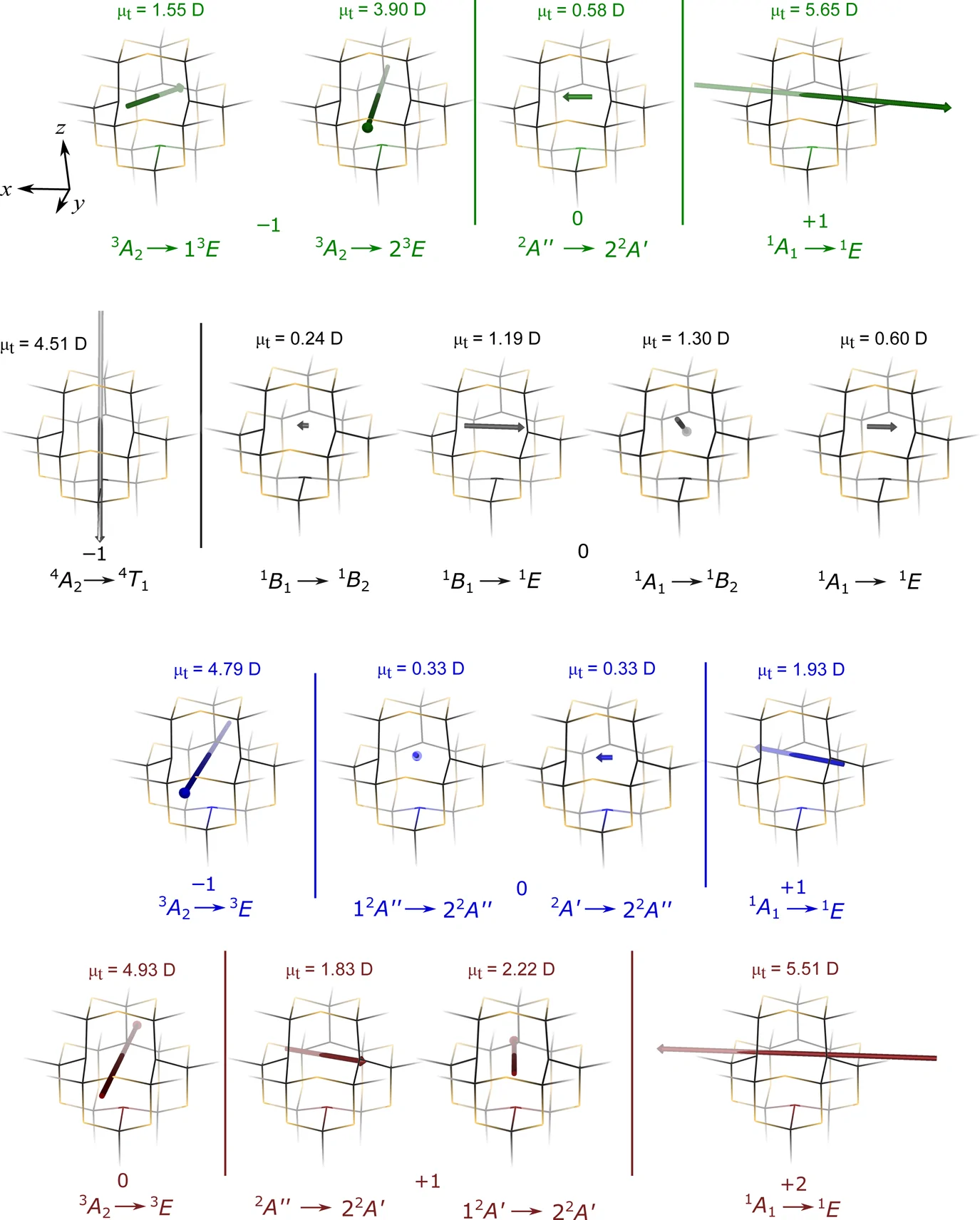
Transition dipole moment vectors. Select electronic excitations (as labeled) within the preferred spin multiplicity in the ground state of all defects with *D*_C_ = B (green), C (black), N (blue), and O (maroon) and their different charges (as shown). For degenerate states, we show only one vector (all transition dipole vector components are tabulated in Table S12). The vectors are shown with the capped clusters and centered approximately in the middle of the C­[3c] atoms (although note that the transition vectors are origin-independent) with length proportional to their magnitude in debye (1 D = 0.208 e·Å). The vectors are defined as *x* = [101̅], *y* = [1̅21̅], and *z* = [111] in the basis of the cubic lattice vectors of diamond.

Figure 4 and Table S12 indicate bright ^3^ *A*_2_ → 1^3^ *E* and even brighter ^3^ *A*_2_ → 2^3^ *E* excitation for B_C_V_C_^–^. Even more intense absorption and eventual emission are expected for ^4^ *A*_2_ → ^4^ *T*_1_ of V_C_^–^ and ^3^ *A*_2_ → ^3^ *E* of N_C_V_C_^–^ and O_C_V_C_^0^. Experiments have shown strong photoluminescence for V_C_^–^ ^86^ and N_C_V_C_^–^,^16^ with no definitive data available for B_C_V_C_^–^ and O_C_V_C_^0^. For B_C_V_C_^–^, N_C_V_C_^–^, and O_C_V_C_^0^, the **μ**_**t**_s are orthogonal to the *D*_C_–V_C_ axis (Figure 4 and Table S12), consistent with their irreps: in *C*_3*v*_, *E* transforms as the *x*- and *y*-axis if *z* is the rotational axis. For V_C_^–^, the three ^4^ *A*_2_ → ^4^ *T*_1_ **μ**_**t**_s point toward three equivalent orthogonal axes (Figure 4 (only one shown) and Table S12). The neutrals and O_C_V_C_^+^, which have broken (thus lower) symmetries, exhibit smaller **μ**_**t**_s than the anions and O_C_V_C_^0^ and are thus expected to be dark to dim emitters. The **μ**_**t**_s are also generally orthogonal to the *D*_C_–V_C_ axis (Figure 4 and Table S12). For V_C_^0^, they are either approximately parallel or orthogonal to a mirror plane. Experimentally, it has been shown that N_C_V_C_^0^ is a dark state of NV centers, consistent with the calculated **μ**_**t**_, which is much smaller than that of N_C_V_C_^–^.^90^ For B_C_V_C_^+^ and O_C_V_C_^2+^ ^1^ *A*_1_ → ^1^ *E*, the **μ**_**t**_s are once again orthogonal to the *D*_C_–V_C_ axis and possess the largest magnitude relative to the other charge states, indicating brightest transitions. N_C_V_C_^+^^1^ *A*_1_ → ^1^ *E* exhibits a **μ**_**t**_ in between those of the anion and neutral defect and includes non-negligible N_C_–V_C_ axial contributions (Figure 4 and Table S12).

### Promising Alternatives to NV^–^ Centers with Suitable Electronic Structures

A viable qubit must have a net spin moment, which makes it susceptible to magnetic manipulation, e.g., by a static magnetic field. The qubit therefore has either a ground state or an accessible (although higher in energy) spin polarized state. Qubit read-out typically involves photoexcitation with a laser and measurement of its eventual fluorescence. Therefore, a qubit must have an absorption and emission profile with energies below the fundamental gap of the host (here, 5.5 eV for diamond). It is also desirable for the qubit to have a shelving state, i.e., a different spin state that makes the qubit momentarily undetectable because of a different fluorescence wavelength, that is relatively long-lived (spin forbidden relaxation back to the ground state of the dominant spin multiplicity). A shelving state stores quantum information longer, potentially long enough to perform a quantum “operation”. If the shelving state is a spin-singlet, then the state is also unperturbed by a static magnet field. The shelving state is accessed, for example, via ISC from the excited state of the dominant spin multiplicity.

B_C_V_C_^+^, V_C_^0^, N_C_V_C_^+^, and O_C_V_C_^2+^ all have spin-singlet ground states, making them unresponsive to a magnetic field. B_C_V_C_^+^, N_C_V_C_^+^, and O_C_V_C_^2+^ have their ground spin-triplet states between the ground and first excited spin-singlet. Unlike the other three, the ground-state spin-triplet of V_C_^0^ (1^3^ *A*_2_) is very close in energy to the symmetry-broken ground-state spin-singlets (^1^ *B*_1_ and ^1^ *A*_1_). For these defect charge states, the spin-triplet is metastable and thus potentially may be used as a quantum bus for longer-term data storage when combined with nuclear spin (having no electron spin in its resting state means no nuclear spin decoherence originating from electron spin), where a transient spin-triplet is optically activated, similar to, e.g., the ST1 color center.^71^ B_C_V_C_^0^, N_C_V_C_^0^, and O_C_V_C_^+^ are all spin-doublets, but their respective spin-quartet states (possible shelving states) have a lowest energy state that is too low in energy or a higher energy state that is too high energy relative to the second excited doublet state, making ISC very unlikely (Figure 3). These defects are also predicted to be dim or dark, making them unsuitable for the fast optical read-out necessary for optically detected magnetic resonance measurements (Figure 4 and Table S12).

We now look closer at B_C_V_C_^–^, V_C_^–^, and O_C_V_C_^0^ and compare them against N_C_V_C_^–^, an already functional qubit. Qubit read-out involves photoexcitation (of the ground-state spin) with a laser and measurement of fluorescence. The intensity of absorption and emission is proportional to the square of the transition dipole moment (**μ**_**t**_). Figure 5A summarizes and plots the magnitude of **μ**_**t**_ with the corresponding VEE of the excitation for these defects in their most preferred (high-spin) multiplicity. Note that for B_C_V_C_^–^, we evaluated the higher energy 2^3^ *E* state because it is brighter and nearly degenerate to an excited-state spin-singlet (2^1^ *E*), which would be suitable for ISC leading to “shelving”, whereas 1^3^ *E* is not (^1^ *A*_1_ is lower by 0.9 eV). As stated in the previous section, **μ**_**t**_s for these defects are in the range of 3.5–5 D (Figure 5A), which makes these excitations have bright emissions (strongly allowed). Accordingly, we plot in Figure 5B the magnitude of **μ**_**t**_ with the corresponding VEE of the excitation of the less favored (low-spin) multiplicity of these defects. We find the low-spin multiplicity radiative emissions to be nearly as bright as the high-spin states except for those of B_C_V_C_^–^.

**Figure 5:**
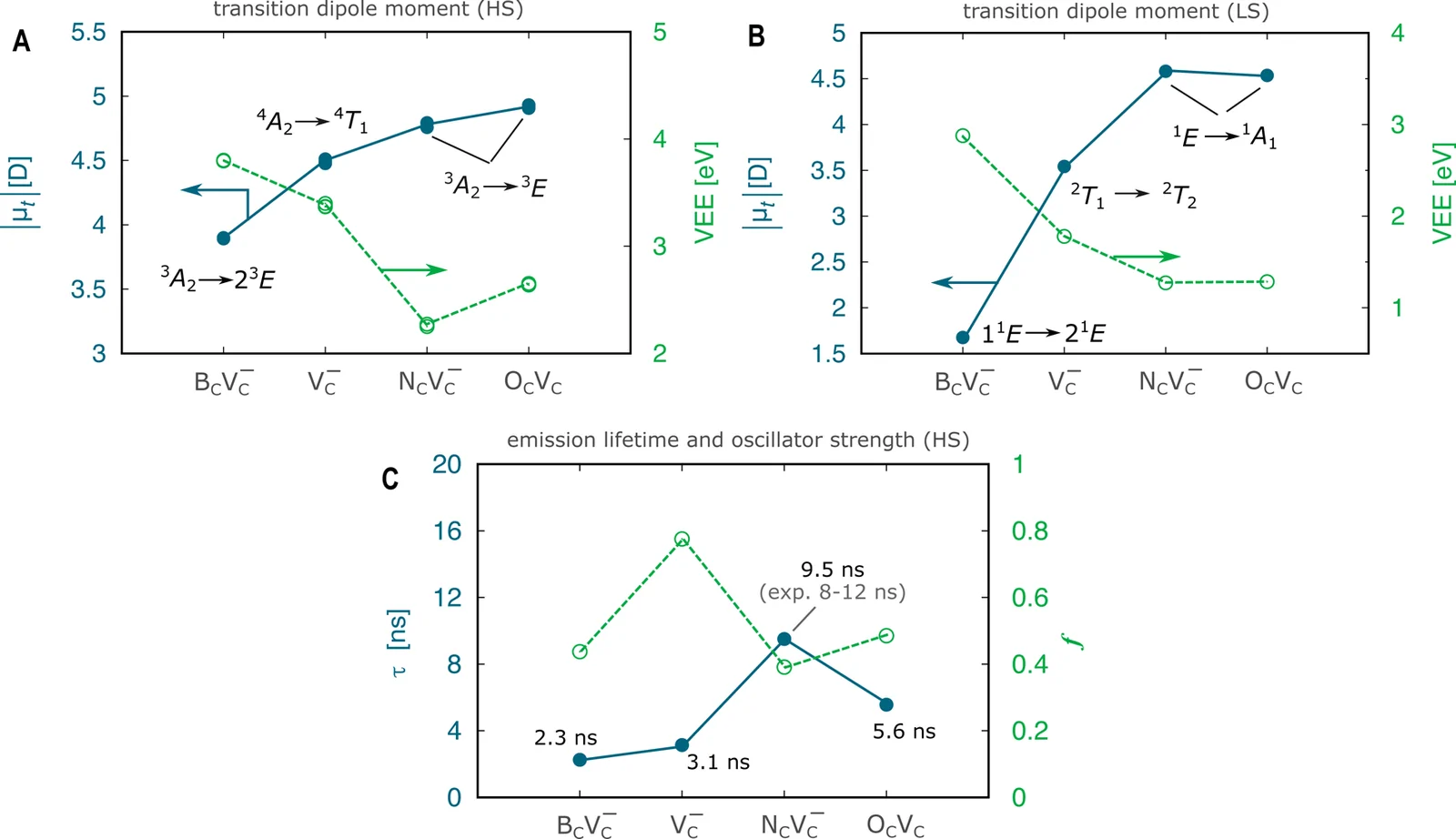
Calculated transition dipole moment magnitudes, emission lifetimes, and oscillator strengths of valence excited states for B_C_V_C_^–^, V_C_^–^, N_C_V_C_^–^, and O_C_V_C_^0^. (A) Transition dipole moment magnitude (|**μ**_**t**_|) from the ground to the second (B_C_V_C_^–^) or first (V_C_^–^, N_C_V_C_^–^, and O_C_V_C_^0^) excited state of the high-spin (HS) state plotted with the vertical excitation energy (VEE). (B) The same plot as (A) but for the corresponding low-spin (LS) states as labeled. (C) Spontaneous emission lifetime (τ) and oscillator strength (*f*) of the HS excited state in (A). Range of experimental values of (*m*_s_-dependent) τ for N_C_V_C_^–^ taken from ref 98 which is matched well by prediction. All transition dipole vector components and *f* values are tabulated in Table S12.

We also calculated the excited states’ natural lifetimes due to relaxation via spontaneous emission (τ) and oscillator strength (*f*), which we plot in Figure 5C. Einstein coefficient *A* relates **μ**_**t**_ and excitation energy with τ, where τ is inversely proportional to |**μ**_**t**_|^2^ and the cube of the excitation energy (Methods, Excited state properties).^95^ Oscillator strength is directly proportional to both |**μ**_**t**_|^2^ and the excitation energy. The absorption cross section is directly proportional to *f*, and the former can be calculated directly from *f* if the spectral line shape is concurrently known.^82,95^ An excited state’s natural lifetime (spontaneous emission) serves as an upper bound because of other faster nonradiative relaxation channels, such as phonon-induced scattering, that may compete. Figure 5C shows that the excited spin-triplets of B_C_V_C_^–^, V_C_^–^, and O_C_V_C_^0^ are shorter-lived than that of N_C_V_C_^–^ by either a third or a half. A shorter-lived excited state means more frequent signal generation, which is thus desirable for read-out. Using *f* as a measure of brightness, 2^3^ *E* B_C_V_C_^–^ and ^3^ *E* O_C_V_C_^0^ are nearly as bright as ^3^ *E* N_C_V_C_^–^. We also found that for B_C_V_C_^–^, 2^3^ *E* is ∼8 times brighter than 1^3^ *E* (*f* [2^3^ *E*]/*f* [1^3^ *E*] = 0.44/0.056 = 7.8, Table S12). The high *f* for ^4^ *T*_1_ V_C_^–^ is in part due to the triple degeneracy of the excited state ^4^ *T*_1_ and nondegenerate ground state ^4^ *A*_2_, allowing for more avenues for excitation. This means stronger light absorption of ^4^ *A*_2_ than emission of ^4^ *T*_1_ in V_C_^–^. B_C_V_C_^–^ and O_C_V_C_^0^ indeed share qualitatively similar properties with N_C_V_C_^–^ ^70,96,97^ but present alternative absorption/emission frequencies and excited-state lifetimes if desired.

## Conclusions

Here, we simulate a series of diamond defects: a carbon vacancy (V_C_) and substitutions (B, N, and O atoms at a C lattice site or B_C_, N_C_, and O_C_, respectively) that are adjacent to a V_C_ to evaluate their magnetic and optical properties. These defects have localized electronic structures, which we model as V_C_-centered molecules embedded in an optimized effective potential, which we derived quantum mechanically from a defective periodic diamond within capped-DFET. We applied molecular orbital principles, an embedded cluster method, and the multiconfigurational *n*-electron second order perturbation theory to computationally characterize the above-mentioned defects for a multitude of spin, charge, and excited states. We found spin-triplets B_C_V_C_^–^ and O_C_V_C_^0^ to be promising alternatives to the well-known N_C_V_C_^–^. Similar to N_C_V_C_^–^, both B_C_V_C_^–^ and O_C_V_C_^0^ have accessible spin-singlets that may luminesce infrared and violet light, respectively, which may act as long-lived magnetically inactive “shelving” states. O_C_V_C_^0^ is valence isoelectronic to N_C_V_C_^–^ but O_C_V_C_^0^ has a lower energy doubly occupied frontier *a*_1_ orbital (with also smaller dopant 2*p* character). The lower *a*_1_ energy raises the first excited triplet state energy *a*_1_ → *e* of O_C_V_C_^0^, i.e., from said *a*_1_ to the singly occupied doubly degenerate *e* orbitals (both almost exclusively derived from the nonbonding 2*sp* ^3^ orbitals of the C atoms at the V_C_). B_C_V_C_^–^, in contrast, has an empty B 2*p*-derived *a*_1_ state immediately above its frontier singly occupied *e* orbitals. Excitation between these states (*e* → *a*_1_) would lead to a less bright fluorescence. The brighter transition of B_C_V_C_^–^ is its second triplet excited state, which is similar in character as the first excited states of N_C_V_C_^–^ and O_C_V_C_^0^. By using our embedded molecular analogue framework for solid-state defects, we were able to quantitatively reproduce a plethora of experimental optoelectronic properties of *D*_C_V_C_ defects.

A full assessment of qubit viability requires looking into the effects of electron–phonon coupling, ground- and excited-state zero-field splitting, intersystem crossing probability into and phosphorescence from the shelving states, and, importantly, quantum coherence lifetimes (and their sensitivity to local magnetic or electric fields). Electronic structures, symmetry, VEEs, transition dipole moments, and excited-state lifetimes provide an initial valuable set of insights into the qubit operation to help screen for or understand the operation of candidate qubits. Ultimately, we showed that a chemistry principle-based analysis of electronically isolated solid-state defects has the potential to accelerate this endeavor.

## Supplementary Material



## Notes

Methods; figures of structural models, embedding potentials, spin density, active space natural orbitals, local structures and relative energies of the symmetry-constrained B_C_V_C_^0^, V_C_^0^, N_C_V_C_^0^, and O_C_V_C_^+^, electronic configurations of higher energy spin multiplicities, charge density differences; and tables of bulk lattice constants of pure and defective diamond, relative energy of the high- and low-spin multiplicities, comparison between local distortions from DFT-r^2^-SCAN-L- and DFT-HSE06-optimized atomic structures for select defects, residual of symmetry operations, character tables, electronic configurations of the ground and excited states, transition dipole moments, lifetimes, and oscillator strengths (PDF)
