# Supplementary material for: A Chemist’s Guide to Solid-State Qubits: Diamond Color Centers as Embedded Vacancy-Centered Molecules
Source: ACS Cent Sci. 2026 Jul 27;12(10):1578–91. doi: 10.1021/acscentsci.6c00548 (PMC13720918; doi:10.1021/acscentsci.6c00548)
Supplement: Supplementary file 1 [file oc6c00548_si_001.pdf]

Supplementary Information

A Chemist's Guide to Solid-State Qubits: Diamond Color Centers as Embedded Vacancy-Centered Molecules

John Mark P. Martirez
Applied Materials and Sustainability Sciences, Princeton Plasma Physics Laboratory, Princeton, New Jersey, 08540-6655, United States
*martirez@pppl.gov, martirez@princeton.edu

Methods

Periodic DFT calculations. We performed spin-polarized Kohn-Sham DFT calculations subject to PBCs within the Vienna Ab-Initio Simulation Package (VASP) version 6.4.[1, 2] We used the projector augmented-wave (PAW) method with frozen core approximation in conjunction with a planewave basis set having a kinetic energy cut-off of 660 eV and standard VASP PAW potentials for all elements (the 1s state is frozen).[3] We used the Gaussian smearing method (0.01 eV width) for the reciprocal space integration along with Γ-point-centered 3×3×3 *k*-point sampling mesh. To simulate (periodic) $D_C V_C$ defects, we remove a C atom and for $D \neq$ C, replaced another (one of the four C atoms adjacent to the vacancy) along the [111] direction with atom *D* in a pristine 128-atom (4×4×4) supercell generated from the two-atom primitive face-centered cubic unit cell (**Fig. S1**, left panel). To generate charged defects, we either add or remove an electron(s) and utilized a uniform compensating background charge to yield an overall neutral supercell. We showed previously that DFT-r^2-SCAN-L reproduces well the experimental lattice constant of pure diamond,[4] and here we show that DFT-HSE06 incurs higher error in lattice constant prediction (**Table S1**) although it provides better baseline electronic structure for ECW (vide infra).[4] We thus optimized these structures within DFT-r^2-SCAN-L without imposing any symmetry except for time-reversal in reciprocal space (**Table S2**).

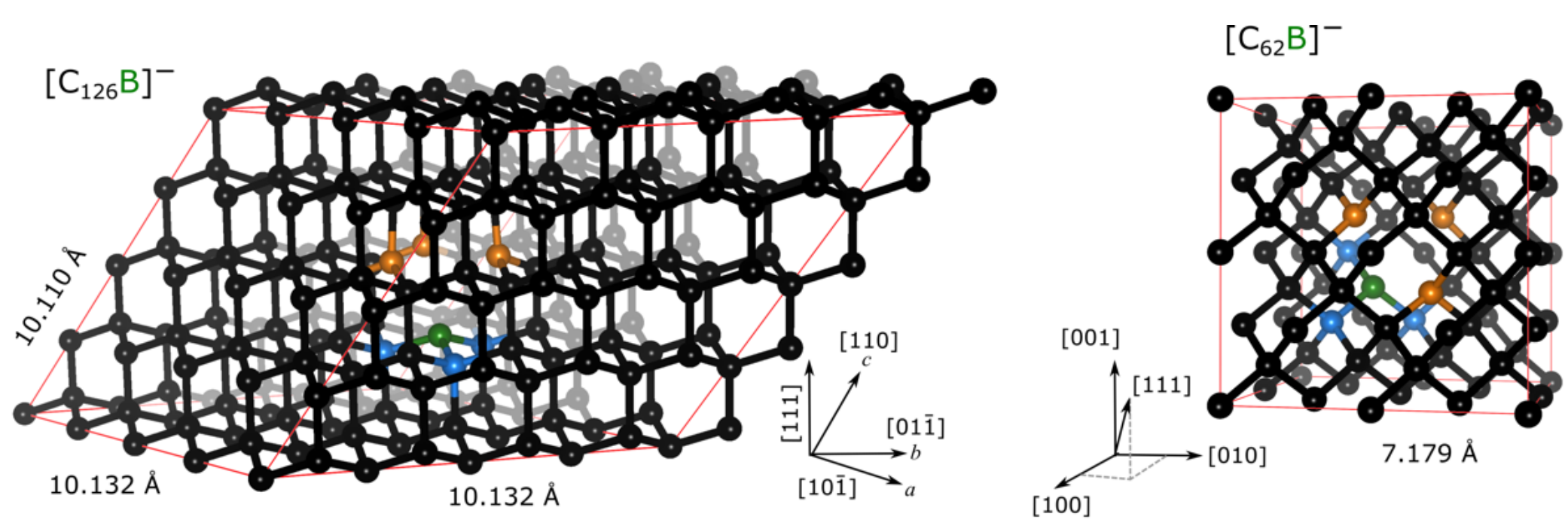

Fig. S1. Periodic defect models with $B_C V_C^-$ defect given as an example. $C_{126}B^-$ and $C_{62}B^-$ (as labelled), optimized within DFT-r^2-SCAN-L. The dimensions of the supercell are given (if only one is given, then all sides are equal). B – green; C – black. For clarity, the three C atoms adjacent to V_C (C[3c]) are colored orange whereas the atoms bound to the B atom (C[n*D*]) are light blue. The red lines mark the supercell edges. The lattice vectors according to the primitive cubic cell are shown. The lattice constants for all $C_{126}D$ structures for different charge states at their respective ground-state spin are reported in **Table S2**.

To determine the preferred ground-state spin of the different defect charge states, we calculated two spin structures for each defect and charge within DFT-HSE06. We did so by using defective 63-atom (2×2×2) supercells generated from the eight-atom conventional simple cubic unit cell (**Fig. S1**, right panel), which were also structurally optimized within DFT-r^2-SCAN-L for each spin configuration (**Fig. S4** shows the real-space spin densities). The energy differences between the different spin states are summarized in **Table S3** and **Note S1**. The DFT-HSE06-predicted ground state spins for the 63-atom supercells were subsequently used for the larger 127-atom, i.e., $C_{126}D$, supercells (vide supra) and thus was also used in the construction of the optimized embedding potential – see *Embedding and fragmentation scheme*. We tested the difference between DFT-r^2-SCAN-L- and DFT-HSE06-predicted

defect-induced distortions as measured by the C[3c]-C[3c] and C[3c]-*D* distances (illustrated in the upper panels of **Fig. 3** in the main text) using the 63-atom (2×2×2) supercells. In DFT-HSE06 optimization of the defects, we evaluated the exact-exchange kernel only at the Γ point for computational feasibility. **Table S4** shows that when using the DFT-r^2-SCAN-L-optimized lattice constants, the two XC functionals only differ in the calculated nearest C[3c]-C[3c] distances at the defects to within 0.01 Å or by less than 0.5%. For C[3c]-*D*, the deviations can be larger (0.009, 0.023, 0.038 Å) but only by at most 1.4%.

Embedding and fragmentation scheme. Capped-DFET[5] is a modification of DFET introduced by Martirez and Carter to account for electron-pair sharing and directional nature of covalent bonds. Within this formalism, when covalent bonds are broken during fragmentation, which gives rise to open-shell atoms (unpaired electron), valence complementary "capping" atoms are introduced to cap the dangling bonds. This is in the same spirit as the link-atom method implemented within QM/Molecular Mechanics (QM/MM) methods[6] predominantly used to model large organic molecules. One of the critical differences between capped-DFET and link-atom QM/MM however is that an effective potential (or embedding potential) is optimized in the former to account for the interaction between the fragments, which is added to the fragments' electronic Hamiltonians. Furthermore, the full system is also described within a QM method, i.e., DFT (instead of MM), here, subject to PBCs. One then calculates energies and other electronic properties in the usual manner using non-periodic capped clusters, with the modified non-periodic electronic Hamiltonian (*Capped-DFET formalism*) within a higher-level theory, e.g., correlated wavefunction (CW) theory, which is now aptly referred to as capped embedded CW (capped emb-CW) theory – see *Excited state calculations*.[4] The nature of the approximation and details of the method are provided in ref. [5].

To simulate D_CV_C defects within capped emb-CW method, we started with the full $C_{126}D$ periodic supercell (**Fig. S2**, top row), we carved out a $C_{15}D$ cluster where the C_{15} includes the three C[3c]s around V_C, the nine nearest neighbors of C[3c]s, and three C[n*D*] (**Fig. S2**, left middle row). The remaining C_{111} thus constitute the environment (**Fig. S2**, right middle row). The $C_{15}D$ cluster contains 12 coordinatively unsaturated C atoms at the fragmentation points. To saturate these C atoms, we add either a F atom when monovalent coordination is required or an O atom when divalent coordination is required, i.e., when the capping element is shared by two C atoms (cap1). At the fragmentation sites of the C_{111} (environment) fragment, the missing coordinating atoms are shared by three C atoms. Therefore, we introduced B atoms at those sites and there are 12 such sites (cap2). This capping scheme produced $C_{15}DF_{12}O_{12}$ and $C_{111}B_{12}$ fragments (**Fig. S2,** middle row), which are coordinatively saturated at the fragmentation points after capping. The auxiliary fragment composed of combined capping elements (cap1 + cap2) from both the cluster and environment ($F_{12}O_{12}B_{12}$) also results in a coordinatively saturated closed-shell hypothetical molecular fragment (**Fig. S2**, bottom row), which is a consequence of valence complementarity principle we abide by in deciding what elements to cap the cluster and environment fragments. We determined $C_{15}DF_{12}O_{12}$ to be a suitable cluster size from a prior study (ref. [4]). In ref. [4] we showed that the defect orbitals in $N_CV_C^-$ originate primarily from the C[3c] and N atoms. The nearest neighbors of these atoms serve to provide the correct local environment to the defect site. The capping atoms provides the medium-range boundary condition that saturates dangling bonds at the edge of the molecules while also preventing charge spill-over into the immediate vacuum space. Finally, the embedding potential ($V_{emb}(\boldsymbol{r})$) tunes the influence of the capping atoms to replicate the crystalline diamond environment (*Capped-DFET formalism*). Ref. [4] also shows that the VEEs calculated using this method weakly depends on the cluster size as well as the size of the supercell from which the cluster was carved.

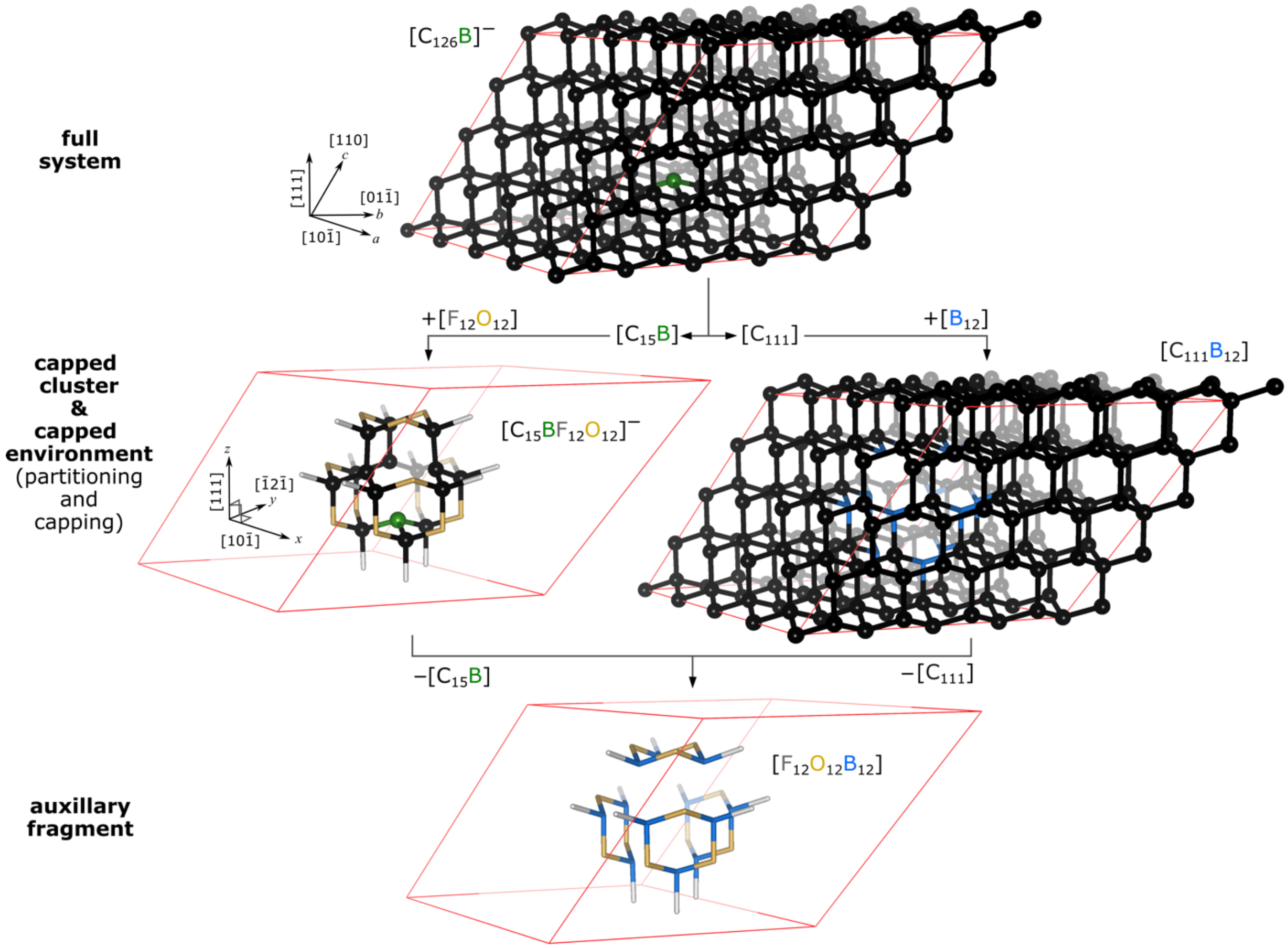

Fig. S2. **Structure partitioning with $B_CV_C^-$ defect given as an example.** Top row: DFT-r^2-SCAN-L-optimized $C_{126}B$ bulk $B_CV_C^{1-}$ color center (lattice constants for all studied defects in **Table S2**). Left middle row: negatively charged molecular $C_{15}BF_{12}O_{12}$ capped cluster. Right middle row: neutral $C_{111}B_{12}$ capped environment. In both fragments, the capping atoms are positioned at the lattice sites of the atoms they replace (no additional structural relaxation performed). Here, the defect (green) and capping (light blue) B atoms are colored distinctively. Bottom row: the auxiliary fragment composed of the capping elements of the cluster and environment fragments, which when together form a closed-shell neutral molecule. The V_{emb}, i.e., effective potential representing the influence of the environment to the cluster and vice versa not captured by the fragments' respective capping elements are shown in **Fig. S3**.

Capped-DFET formalism. The interaction potential, i.e., embedding potential or $V_{emb}(\boldsymbol{r})$, is optimized within DFT via the maximization of the extended Wu-Yang functional,[7] W

$$W = E_{DFT}^{cl+cap1}[\rho^{cl}, V_{emb}] + E_{DFT}^{env+cap2}[\rho^{env}, V_{emb}] - \int V_{emb}\,(\rho^{full} + \rho^{cap1+cap2})\,dr \qquad \textbf{Eq. S1}$$

with a gradient given by

$$\frac{\delta W}{\delta V_{emb}(\boldsymbol{r})} = \rho^{cl+cap1}(\boldsymbol{r}) + \rho^{env+cap2}(\boldsymbol{r}) - \rho^{full}(\boldsymbol{r}) - \rho^{cap1+cap2}(\boldsymbol{r}) \qquad \textbf{Eq. S2}$$

where $\rho^{cl+cap1}(\boldsymbol{r})$, $\rho^{env+cap2}(\boldsymbol{r})$, and $\rho^{cap1+cap2}(\boldsymbol{r})$ are the self-consistent electron densities of the cluster (cl) with capping elements 1 ($cap1$), environment (env) with capping elements 2 ($cap2$), and the combined capping elements $cap1$+ $cap2$, *i.e.*, the auxiliary fragment. $V_{emb}(\boldsymbol{r})$ yields the influence of the environment on the cluster and vice versa not provided by the fragments' (cluster and environment) respective capping elements. When **Eq. S1** is maximized, **Eq. S2** becomes zero, therefore,

$$\rho^{cl+cap1}(\boldsymbol{r}) + \rho^{env+cap2}(\boldsymbol{r}) - \rho^{cap1+cap2}(\boldsymbol{r}) = \rho^{full}(\boldsymbol{r}) \quad \textbf{Eq. S3}$$

Eq. S3 shows that after (approximately) removing the contributions of the fragments' respective capping elements via the auxiliary fragment, the sum of the electron densities of the capped cluster and capped environment reproduces the full system electron density. Accordingly, the capped-cluster and capped-environment one-electron Hamiltonians (H^0) were modified as

$$H^0_{emb} = H^0 + V_{emb} \quad \textbf{Eq. S4}$$

For crystalline solids, $V_{emb}(\boldsymbol{r})$ optimization is performed under PBCs. Note that if the supercell is large and the cluster is structurally and electronically distinct from its environment, one may consider the optimized $V_{emb}(\boldsymbol{r})$ to be non-periodic.

Using the above-described capped cluster and capped environment, we performed $V_{emb}(\boldsymbol{r})$ optimization using a modified VASP v. 6.4, which utilizes periodic DFET implementation within the PAW method by Yu, Libisch, and Carter.[8, 9] We performed the $V_{emb}(\boldsymbol{r})$ optimization within DFT-HSE06, which reproduces accurately the electronic structure of pure diamond.[4] To manage computational cost, we down-sampled the reciprocal space to only include the Γ-point in evaluating the exact exchange kernel within DFT-HSE06 for the 128-atom (4×4×4) supercell. **Fig. S3** shows the optimized $V_{emb}(\boldsymbol{r})$ for all defects and charge states at their respective ground-state spin configuration (**Table S3**).

Excited state calculations. We performed all embedded cluster calculations within Molpro version 2024.1.1.[10, 11] We used augmented correlation-consistent polarizable valence double ζ (aug-cc-pvdz)[12] atom-centered Gaussian-type orbital (GTO) basis sets alongside the complementary auxiliary bases for the evaluation of the two-electron integrals. We transformed the VASP-optimized V_{emb} from real-space Cartesian grid to the GTO bases via the embedding integral generator code (EmbeddingIntegralGenerator).[13] When then add the V_{emb} matrix in the GTO basis to the one-electron Hamiltonian. We did not impose any symmetry restrictions in the electronic structure optimization. Most QM codes, including Molpro, could only handle Abelian point groups (e.g., C_1, C_s, C_i, C_{2v}, etc.), where all symmetry operations commute. Hence, even if we wish, we can't impose a non-Abelian point group on the electronic structures. Furthermore, imposition of symmetry usually requires reorientation of the molecule so that, e.g., the main symmetry axis aligns with the Cartesian *z* direction, and a reflection plane is parallel to the Cartesian *xz* plane. This re-orientation is currently not supported in the transformation of V_{emb} from real space to atom-centered basis space, which should be concurrently performed with the molecular reorientation.

We found in a prior study that use of a larger basis set, namely the aug-cc-pv triple ζ (aug-cc-pvtz), leads to only ~ 0.01 eV change in the calculated VEEs of $N_CV_C^-$. We performed spin-pure state-averaged CASSCF (SA-CASSCF), averaging over three or more states, to include static correlation and to calculate for the ground and (a few) valence excited states. Each state is given an equal weight, and we did not impose any symmetry. Each spin multiplicity was calculated using spin-pure configuration state functions (CSFs) in the many-body wavefunction expansion. We performed active space (AS) optimization using the molecular orbital theory as guide to determine the appropriate correlation orbital space (see Main text). Previously we showed that including the occupied V_C-centered orbitals, *i.e.*, orbitals that spatially extend into the vacancy that originate from the C[3c]s and the dopant, and their necessary correlating virtual orbitals yields converged calculated VEEs.[4] Here, we determined an optimal AS size for each defect charge by incrementally adding virtual orbitals into the AS along with the fixed number of occupied V_C-centered orbitals, so long as all the virtual orbitals contribute, i.e., are occupied, in any configuration of any state with absolute configuration interaction (CI) coefficients (c_i) that is greater than or equal to 0.05. For a given defect type and charge, the different spin manifolds may not yield the same optimal AS size. For consistency in correlation space, we use in the end whichever is the larger optimal AS size determined for the spin manifolds for a given defect charge. The optimal AS sizes for SA-CASSCF for the different defects and charges are shown in **Figs. S5-S8**. Finally, we included the dynamic correlation via a multireference second-order perturbation theory method, namely, NEVPT2,[14] which was proven in the literature to be robust in determining excitation energies and relative energies of different spins with no semi-empirical input.[14, 15] We calculated the VEE for the same spin multiplicity as the energy difference between the V_{emb}-embedded capped cluster NEVPT2 energies of the nth excited ($E^{cl+cap1}_{NEVPT2}(n)$) and ground electronic states ($E^{cl+cap1}_{CW}(n=0)$)

$$VEE(0 \rightarrow n) = E^{cl+cap1}_{NEVPT2}(n) - E^{cl+cap1}_{NEVPT2}(n=0) \quad \textbf{Eq. S5}$$

Additional information on the calculation of excited state properties, namely, spin energy splitting, oscillator strength (f), transition dipole moment (μ_t), and lifetime (τ) are described below (*Excited state properties*).

Excited state properties. To calculate the energy difference between two spin manifolds, we performed separately a ground-state SA-CASSCF where we averaged over the two spin multiplicities using the full set of Slater determinants instead of CSFs, which ensures that both spins are described in equal footing. Each spin-manifold received an equal weight, i.e., $\frac{1}{2}$, during self-consistent optimization of the state-average energy. If a spin-manifold is degenerate, each state of said degenerate spin manifold was given a weight of $\frac{1}{4}$ and $\frac{1}{6}$ when degeneracy is 2 and 3, respectively, which ensure that the combined weight of each spin manifold is $\frac{1}{2}$. For a given spin manifold, we also considered all nearly degenerate states in state-averaging, i.e., energy differences < 0.3 eV, which are states whose degeneracy are lost due to broken structural symmetry via Jahn-Teller distortion (C_s and D_{2d}). We followed this with NEVPT2 on each state. We use the energy difference between the two spin multiplicities in their respective electronic ground state to reference the energy differences between their excited states and thus also calculate the singlet-triplet (S-T) or doublet-quartet (D-Q) energy splitting in the excited state.

The transition dipole moment vector ($\boldsymbol{\mu_t}$) and its magnitude is

$$\mu_t = |\boldsymbol{\mu_t}| = \left[\left|\mu_{t,x}\right|^2 + \left|\mu_{t,y}\right|^2 + \left|\mu_{t,z}\right|^2\right]^{\frac{1}{2}} \quad \textbf{Eq. S6}$$

where $\mu_{t,[x,y,z]}$ are the Cartesian components of the transition dipole moment vector, calculated from a multi-state SA-CASSCF. The $\mu_{t,[x,y,z]}$s are origin invariant because all the states are guaranteed to be orthogonal, even though for a charged defect the dipole moment is origin dependent.

The oscillator strength (*f*) for an $i \rightarrow j$ transition is

$$f(i \rightarrow j) = \frac{2}{3}\frac{m_e}{e^2\hbar^2} VEE(i \rightarrow j)|\boldsymbol{\mu_t}|^2 \quad \textbf{Eq. S7}$$

where VEE is the energy for the $i \rightarrow j$ excitation in ha (calculated from emb-NEVPT2), $\boldsymbol{\mu_t}$ is in e·bohr (from the reference SA-CASSCF), and $f(i \rightarrow j)$ is dimensionless. m_e is the rest mass of an electron, e is elementary charge, and $\hbar$ is Planck constant divided by 2π. In atomic units, the constants are simply equal to 1.[16] For multiply degenerate state j with multiplicity m_j, to account for the multiple excitation channels

$$f(i \rightarrow j) = \sum_{n=1}^{m_j} f_n(i \rightarrow j) \quad \textbf{Eq. S8}$$

where $f_n(i \rightarrow j)$s are the oscillator strengths of the individual states n that comprise state j. For (approximately) identical $\boldsymbol{\mu_t}$s, and states i and j with multiplicities m_i and m_j, **Eq. S8** can be generalized as

$$f(i \rightarrow j) = \frac{m_j}{m_i} f_n(i \rightarrow j) \quad \textbf{Eq. S9}$$

Finally, we calculated the natural lifetime (τ), i.e., due to a single relaxation event (spontaneous emission) of the excited states as the reciprocal of the Einstein coefficient[16] for spontaneous emission (A) from state j to lower energy state i

$$\frac{1}{A(j \rightarrow i)} = \tau(j \rightarrow i) = 2.418884 \times 10^{-8} \frac{c^3}{2n_r VEE(i \rightarrow j)^2 f(i \rightarrow j)} \frac{m_j}{m_i} \quad \textbf{Eq. S10}$$

where c is the speed of light in vacuum 137.03604 a.u., n_r is the wavelength-dependent refractive index of the medium (diamond), VEE is in ha, $f(i \rightarrow j)$ from **Eq. S9**, and 2.418884×10^{-8} is the conversion constant from a.u. to ns. We evaluated n_r using the following formula[17] that depends on the wavelength of light (λ) in mm

$$n_r = \sqrt{\frac{4.3356\lambda^2}{\lambda^2 - (0.1060)^2} + \frac{0.3306\lambda^2}{\lambda^2 - (0.1750)^2} + 1} \qquad \textbf{Eq. S11}$$

Eq. S11 is applicable from λ= 225 nm to infrared.[17]

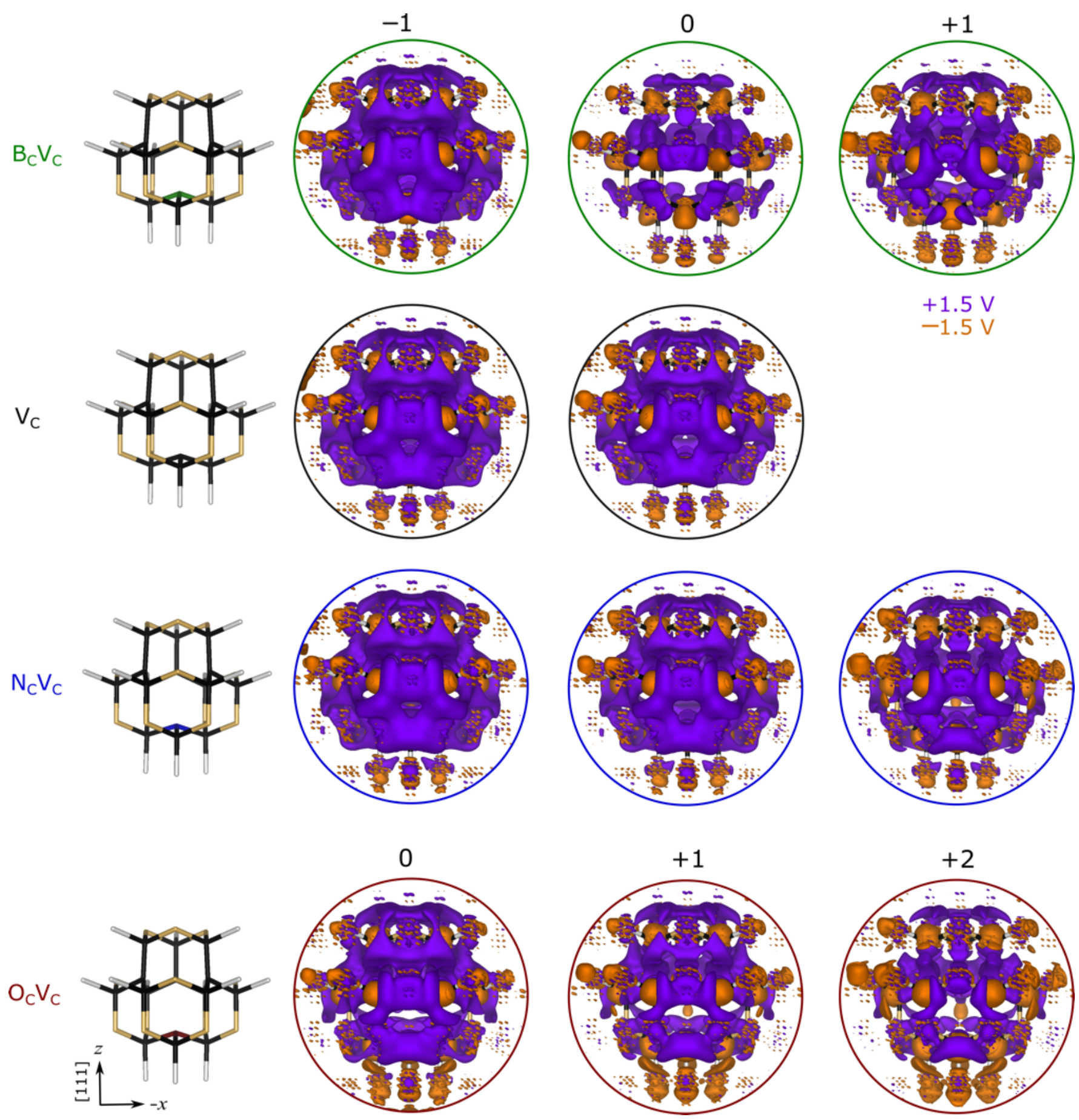

Fig. S3. **Optimized embedding potentials**. DFT-HSE06-optimized V_{emb} for $C_{15}\mathit{D}F_{12}O_{12}$ capped clusters along with $C_{111}B_{12}$ capped environment (**Fig. S2**) for different charges with their corresponding ground-state spin multiplicity (**Table S3**). V_{emb} is free of slowly converging periodic excess charge Coulomb interactions by construction. Purple – repulsive, orange – attractive potential. Vectors: x = $[10\bar{1}]$, y = $[\bar{1}2\bar{1}]$, and z = $[111]$.

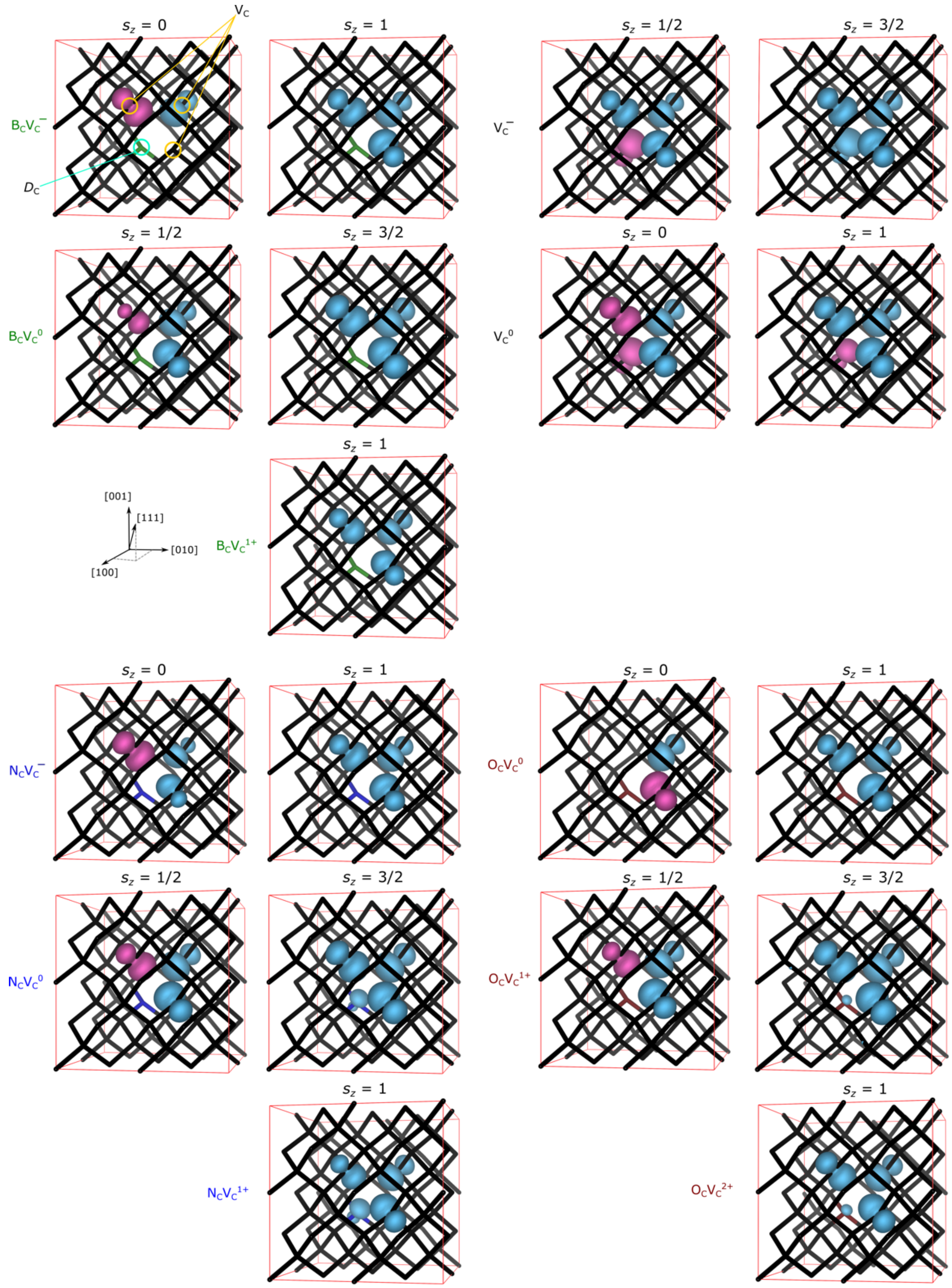

Fig. S4. **DFT-HSE06 magnetic ordering.** Spatial spin-density difference plots for the s_z values in **Table S3** (as labelled) calculated from periodic 63-atom cubic supercells within DFT-HSE06 using structures optimized within DFT-r^2-SCAN-L (spin-symmetric $s_z = 0$ naturally is not shown). Iso-surface: 0.015 e/bohr^3. Excess spin-up – light blue, spin-down – pink.

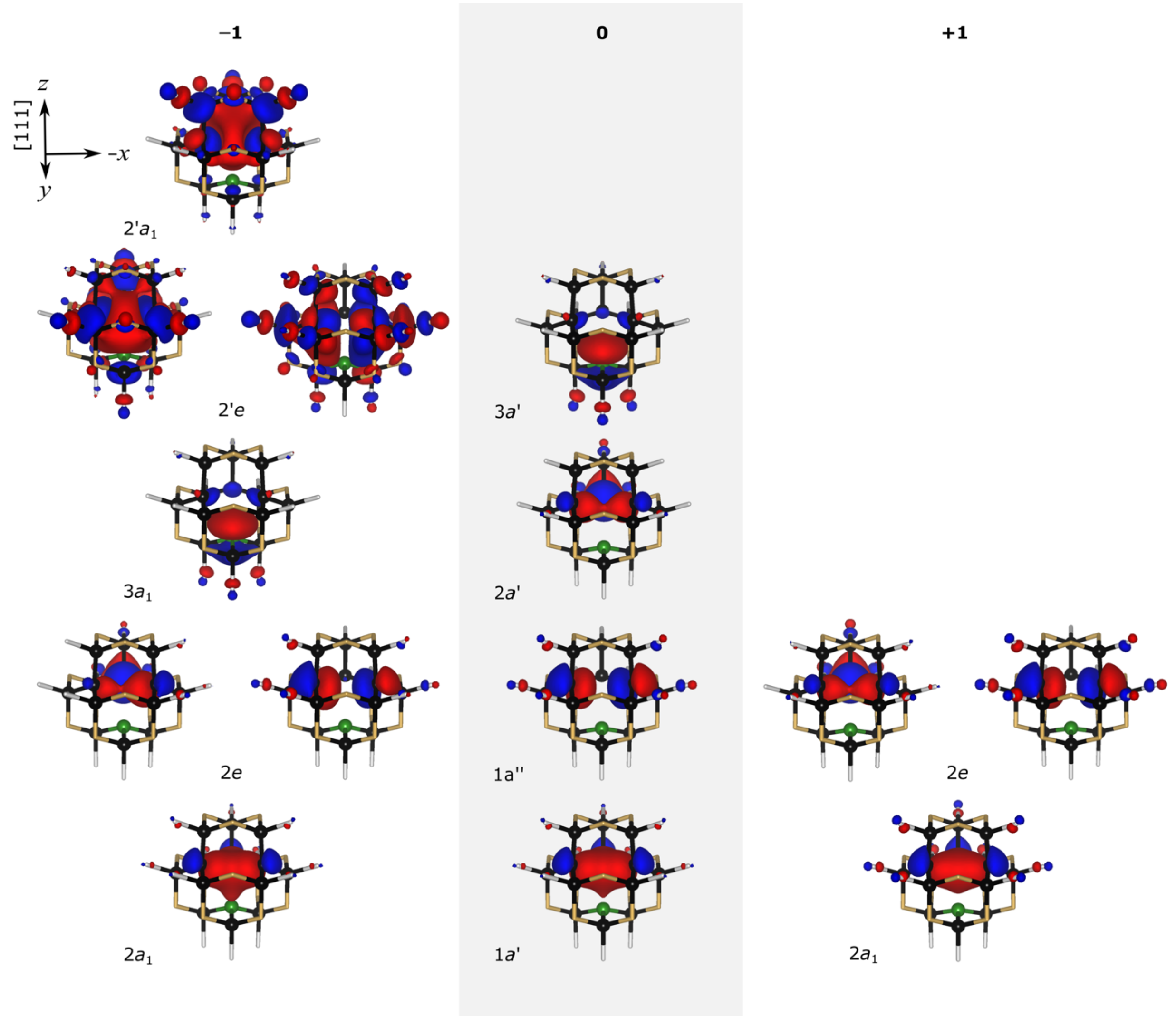

Fig. S5. B_CV_C state-averaged AS natural orbitals for the lowest-energy spin multiplicity. Obtained from diagonalizing the CASSCF *n*-state-averaged density matrix with n = 5, 3, and 3 for charge and spin multiplicity (q,2s+1) = (–1,3), (0,2), and (+1,1), respectively. Orbitals are labelled following C_{3v} Mulliken labels for $\pm$1 and C_s Mulliken labels for 0 as shown in Figs. 2 and 3 in the main text. Isosurface: $\pm$0.06 (e/bohr^3)$^{1/2}$ predominantly occupied orbitals and $\pm$0.03 (e/bohr^3)$^{1/2}$ for the more diffused correlating virtual orbitals (*orbital numerical index* marked with ' in q = –1). The vectors are defined as: $x = [10\bar{1}], y = [\bar{1}2\bar{1}], z = [111]$ in the basis of the cubic lattice vectors of diamond.

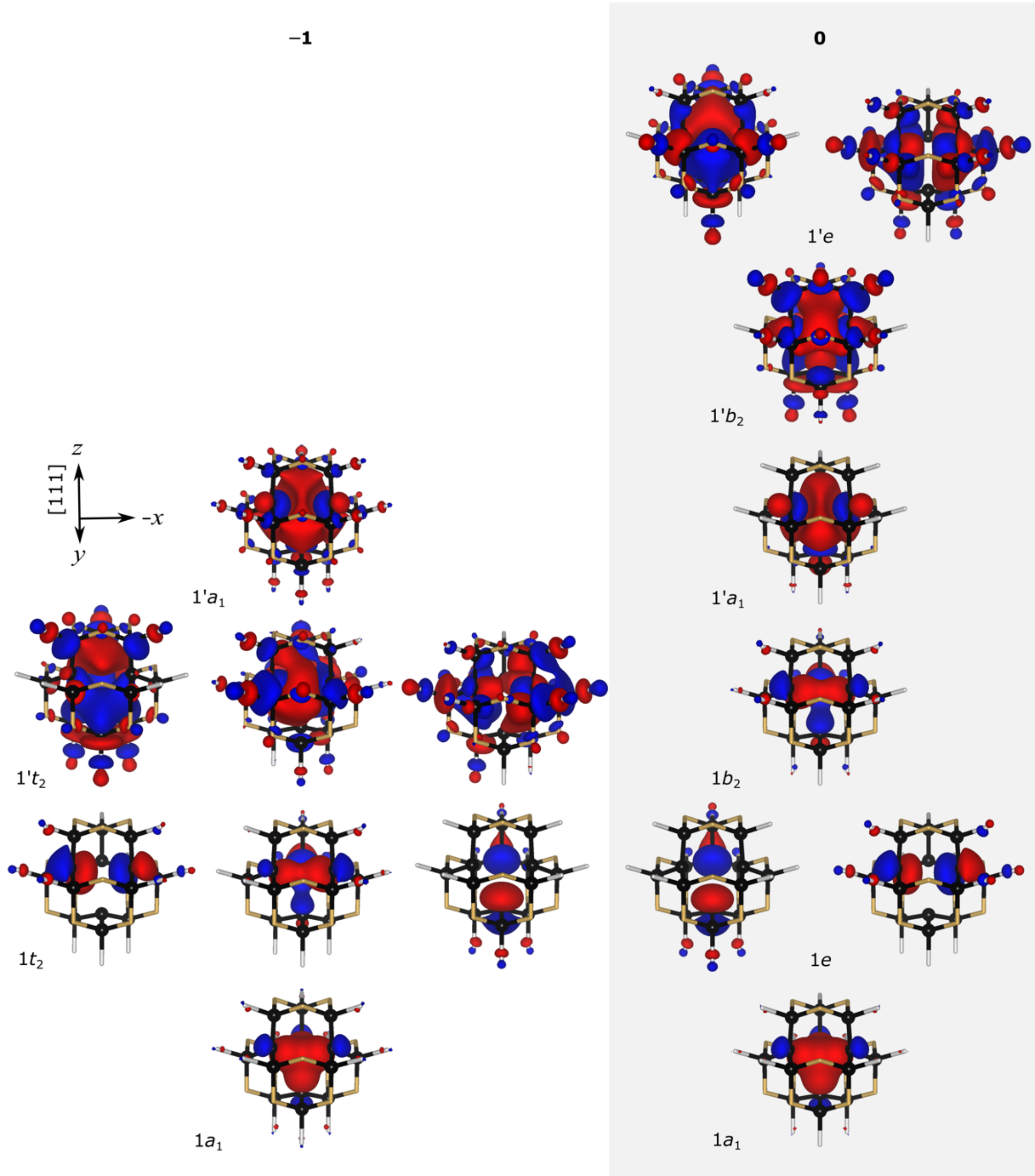

Fig. S6. **V_C state-averaged AS natural orbitals of the ground-state spin multiplicities**. Obtained from diagonalizing the CASSCF *n*-state-averaged density matrix with *n* = 4 and 8 for charge and spin multiplicity (*q*,2*s*+1) = (–1,4) and (0,1), respectively. Orbitals are labelled following T_d Mulliken labels for −1 and D_{2d} Mulliken labels for 0 as shown in Figs. 2 and 3 in the main text. Isosurface: $\pm$ 0.06 (e/bohr^3)$^{1/2}$ predominantly occupied orbitals and $\pm$ 0.03 (e/bohr^3)$^{1/2}$ for the more diffused correlating virtual orbitals (*orbital numerical index* marked with '). The vectors are defined as: $x = [10\bar{1}], y = [\bar{1}2\bar{1}], z = [111]$ in the basis of the cubic lattice vectors of diamond.

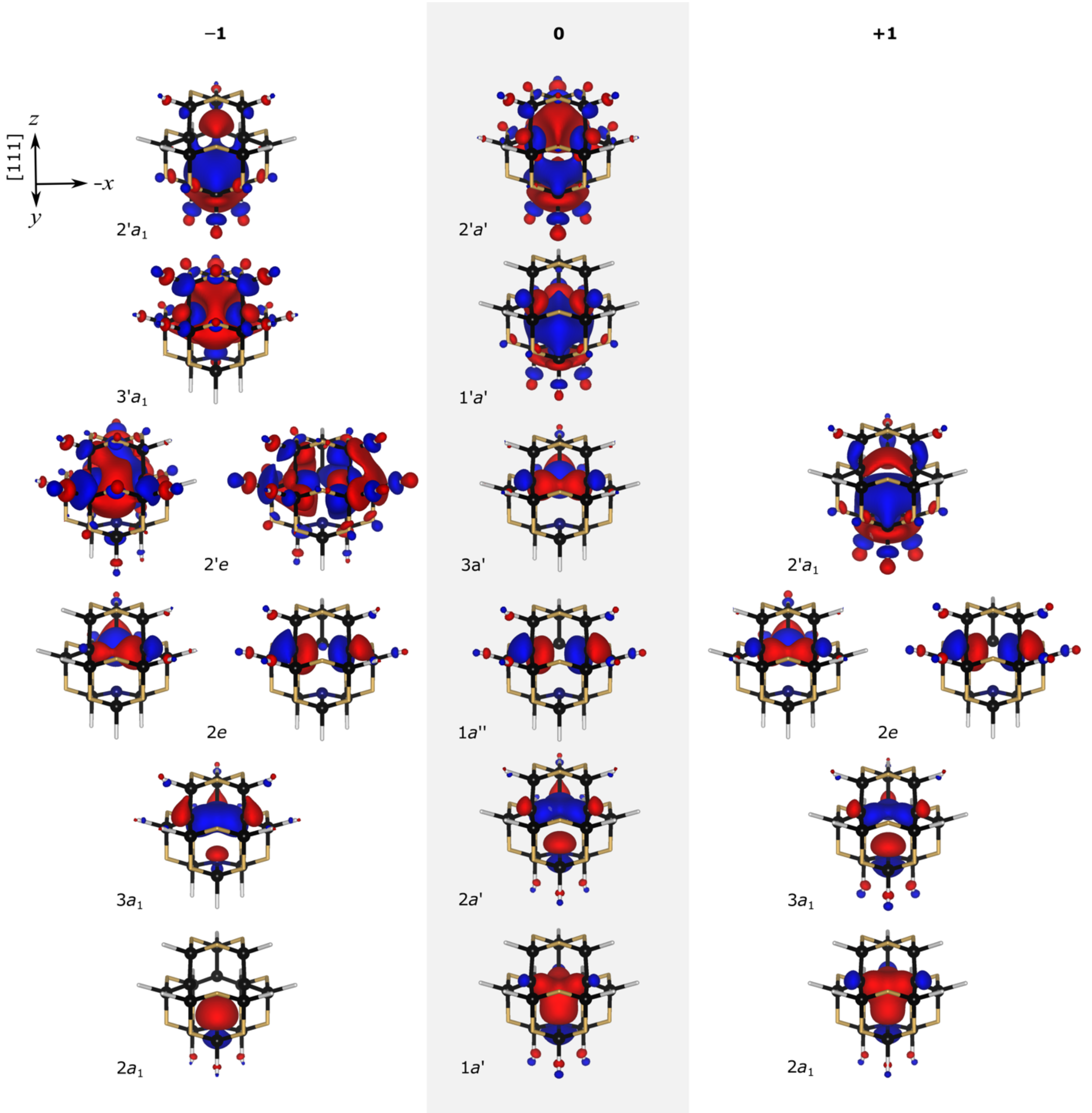

Fig. S7. **N_CV_C state-averaged AS natural orbitals of the ground-state spin multiplicities**. Obtained from diagonalizing the CASSCF *n*-state-averaged density matrix with *n* = 3 for charge and spin multiplicity (*q*,2*s*+1) = (–1,3), (0,2), and (+1,1), respectively. Orbitals are labelled following C_{3v} Muliken labels for ± 1 and C_s Mulliken labels for 0 as shown in Figs. 2 and 3 in the main text. Isosurface: ± 0.06 (e/bohr^3)$^{1/2}$ predominantly occupied orbitals and ± 0.03 (e/bohr^3)$^{1/2}$ for the more diffused correlating virtual orbitals (*orbital numerical index* marked with '). The vectors are defined as: $x = [10\bar{1}], y = [\bar{1}2\bar{1}], z = [111]$ in the basis of the cubic lattice vectors of diamond.

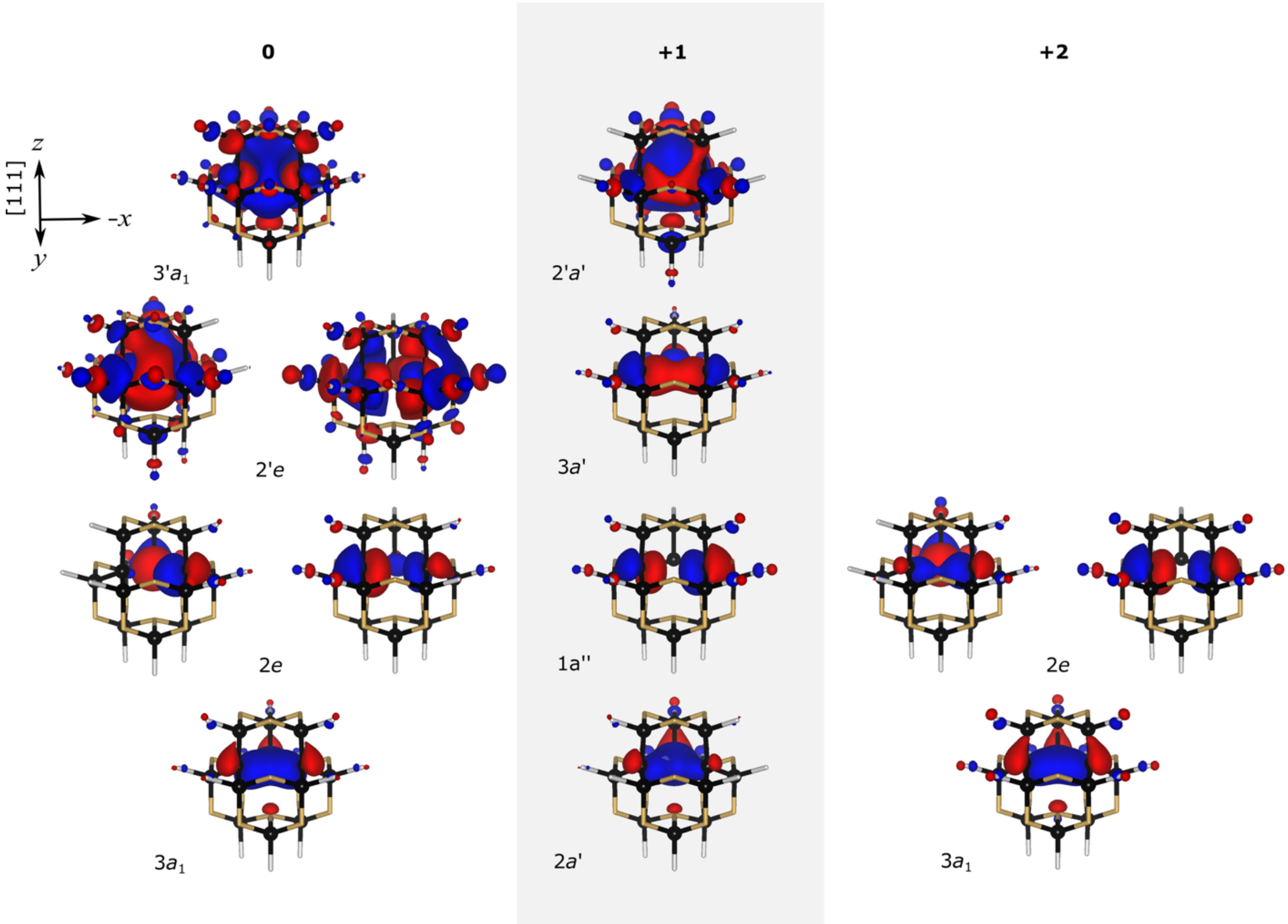

Fig. S8. O_CV_C state-averaged AS natural orbitals of the ground-state spin multiplicities. Obtained from diagonalizing the CASSCF *n*-state-averaged density matrix with *n* = 3 for charge and spin multiplicity (*q*,2*s*+1) = (–1,3), (0,2), and (+1,1), respectively. Orbitals are labelled following C_{3v} Mulliken labels for ± 1 and C_s Mulliken labels for 0 as shown in Figs. 2 and 3 in the main text. Isosurface: ± 0.06 (e/bohr^3)$^{1/2}$ predominantly occupied orbitals and ± 0.03 (e/bohr^3)$^{1/2}$ for the more diffused correlating virtual orbitals (*orbital numerical index* marked with '). The vectors are defined as: $x = [10\bar{1}], y = [\bar{1}2\bar{1}], z = [111]$ in the basis of the cubic lattice vectors of diamond.

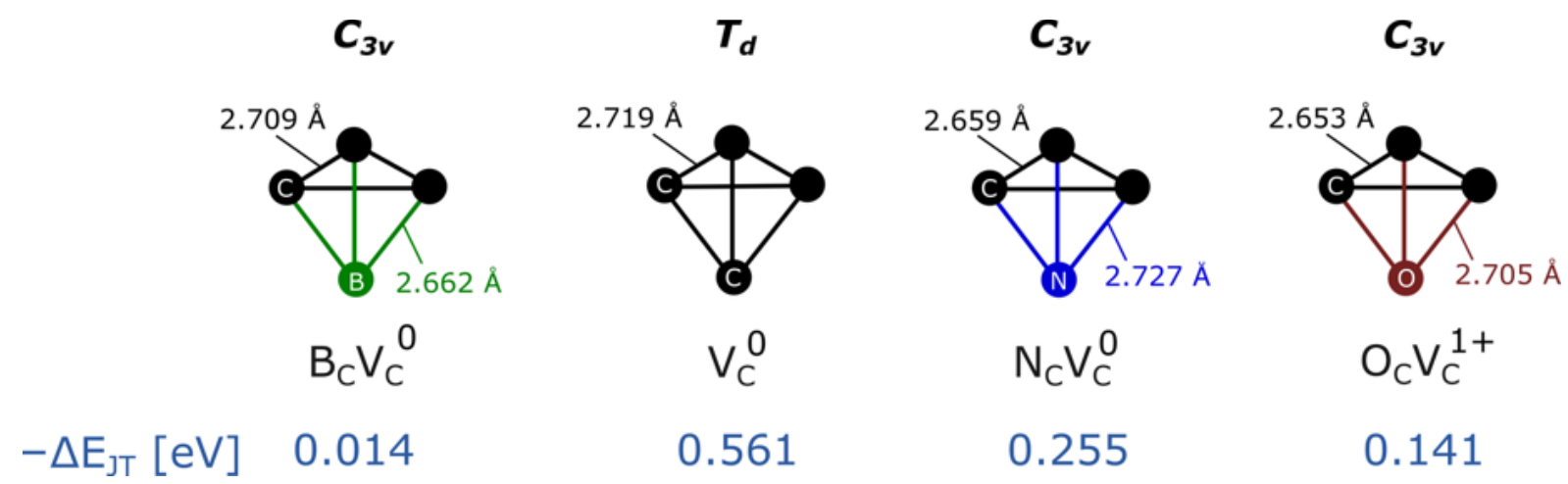

Fig. S9. Local defect structures of symmetry constrained $B_CV_C^0$, V_C^0, $N_CV_C^0$, and $O_CV_C^+$ and the negative of the Jahn-Teller distortion energy ($-\Delta E_{\mathrm{JT}}$). Nearest C[3c]-C[3c] and C[3c]-*D* distances corresponding to C_{3v} ($B_CV_C^0$, $N_CV_C^0$, and $O_CV_C^+$) or T_d (V_C^0) symmetry. The corresponding Jahn-Teller-distorted distances are shown in **Fig. 3** of the main text. $-\Delta E_{\mathrm{JT}}$ was calculated from DFT-r^2-SCAN-L at the defects' ground-state spin multiplicity.

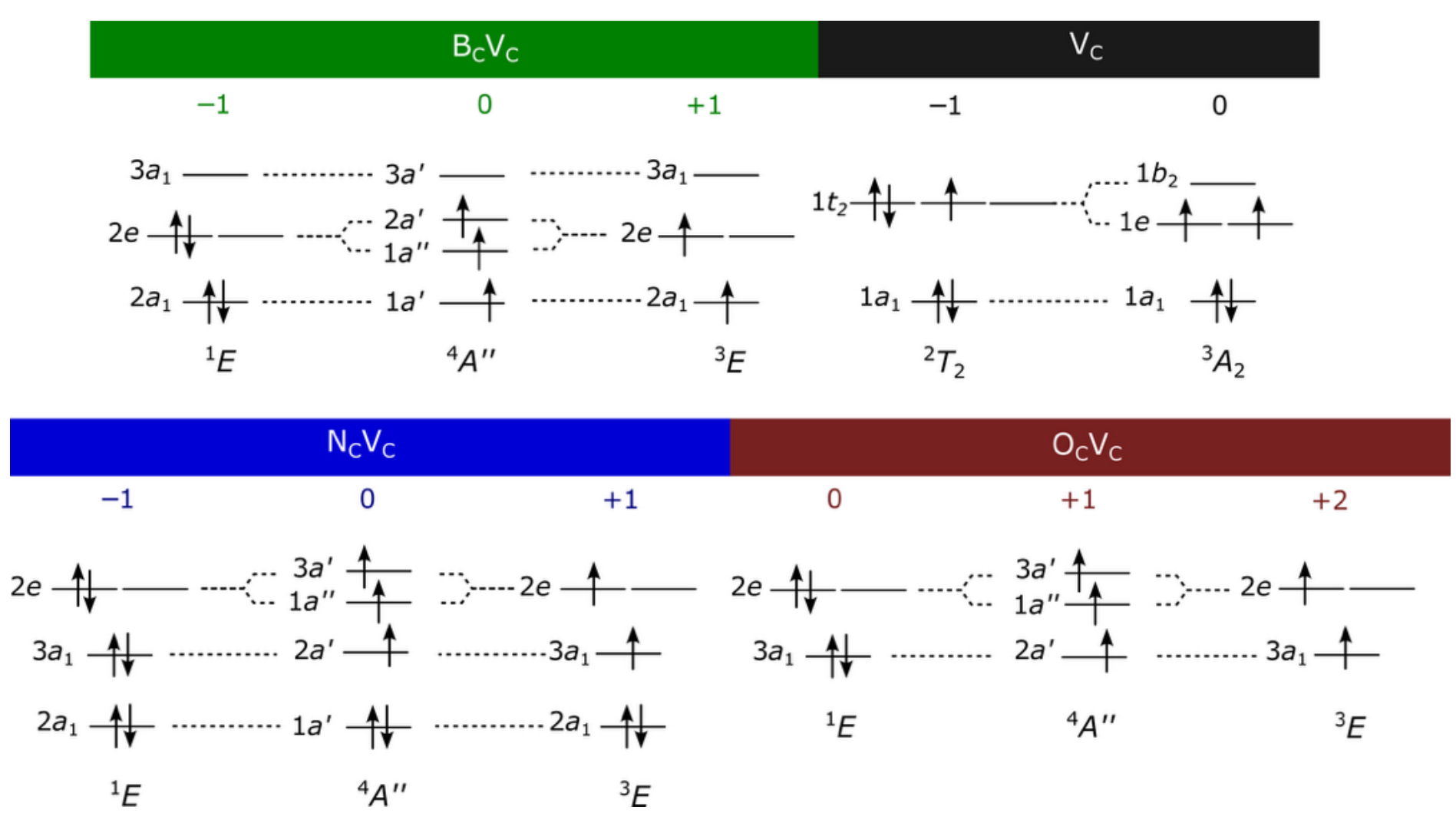

Fig. S10. Representative lowest-energy electronic configuration for the higher-energy spin multiplicity for each dopant and charge. Mulliken labels are shown below each configuration. See **Fig. 3** in the main text for the corresponding lower-energy spin multiplicities.

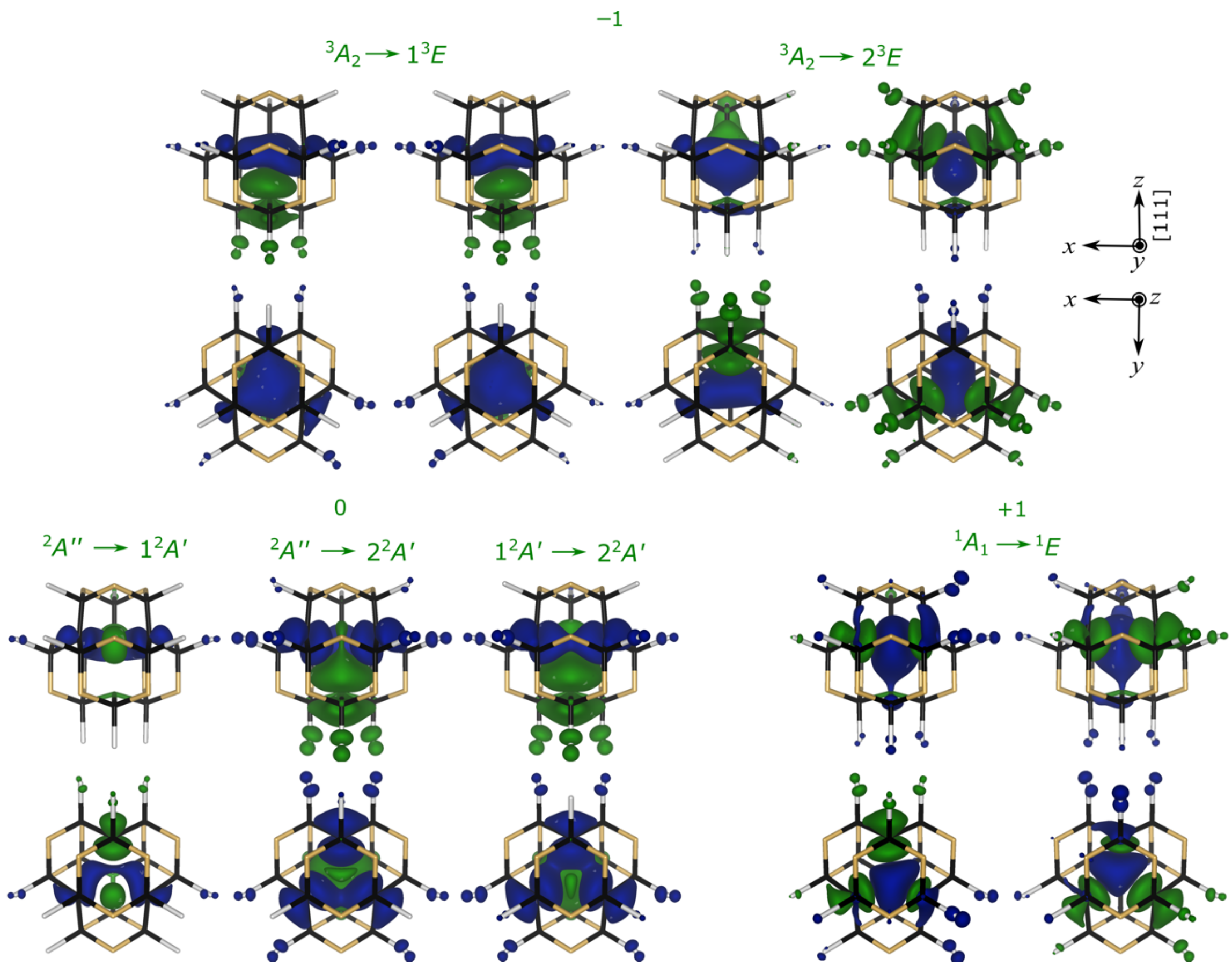

Fig. S11. **B_CV_C excited- and ground-state electron density difference ($\rho_j - \rho_i$) plots**. Densities from SA-CASSCF *n*-state-averaged density matrix (transition type as labelled) with *n* = 5, 3, and 3 for charge and spin multiplicity (*q*,2*s*+1) = (–1,3), (0,2), and (+1,1), respectively. The plots are labelled by excitation type. Isosurface: green (gain)/blue (loss) = $\pm$0.002 e/bohr^3. The vectors are defined as: $x = [10\bar{1}], y = [\bar{1}2\bar{1}], z = [111]$ in the basis of the cubic lattice vectors of diamond.

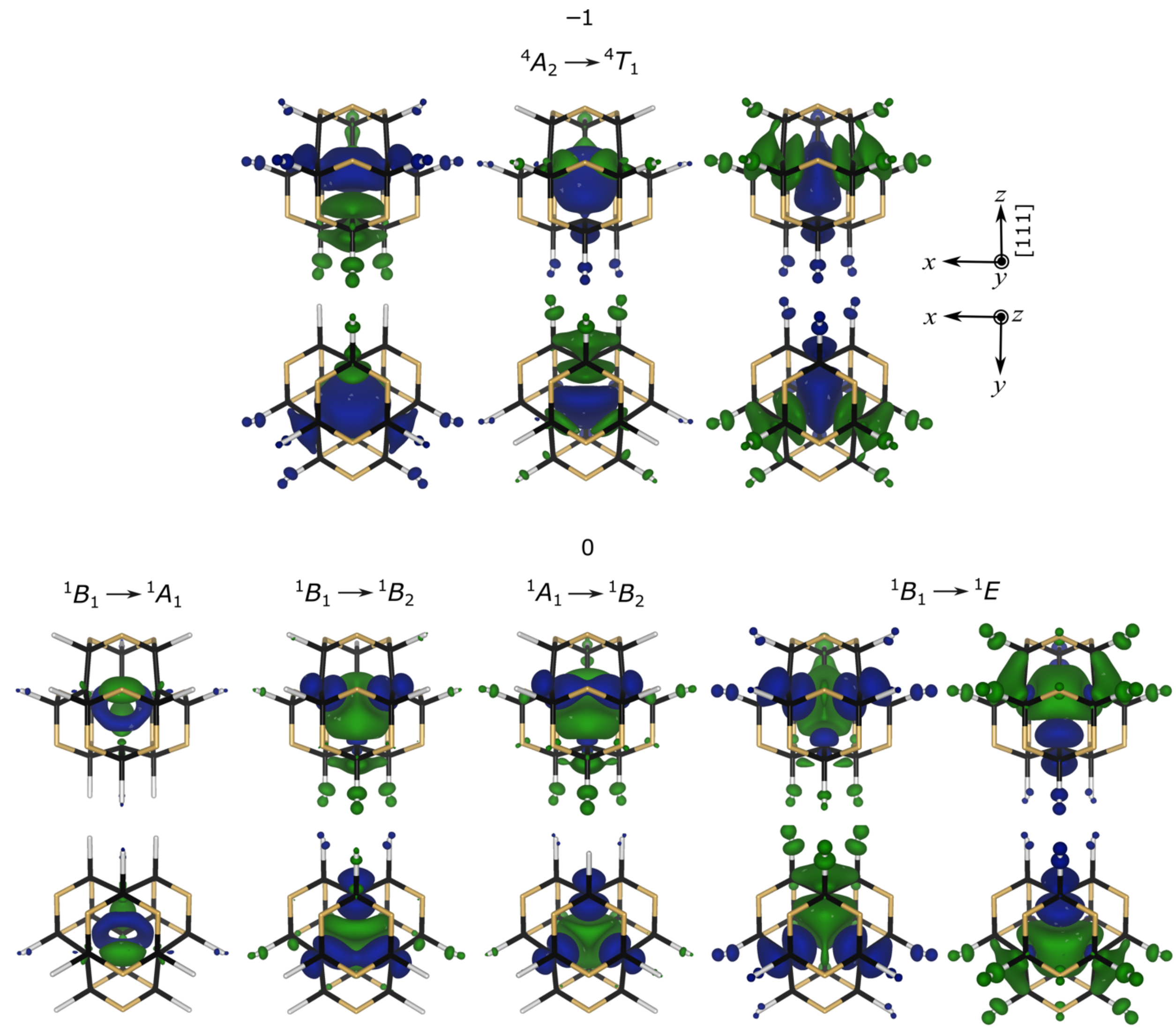

Fig. S12. **V_C excited- and ground-state electron density difference ($\rho_j - \rho_i$) plots**. Densities from SA-CASSCF *n*-state-averaged density matrix (transition type as labelled) with *n* = 4 and 8 for charge and spin multiplicity (*q*,2*s*+1) = (–1,4) and (0,1), respectively. The plots are labelled by excitation type. Isosurface: green (gain)/blue (loss) = $\pm$ 0.002 e/bohr^3. The vectors are defined as: $x = [10\bar{1}], y = [\bar{1}2\bar{1}], z = [111]$ in the basis of the cubic lattice vectors of diamond.

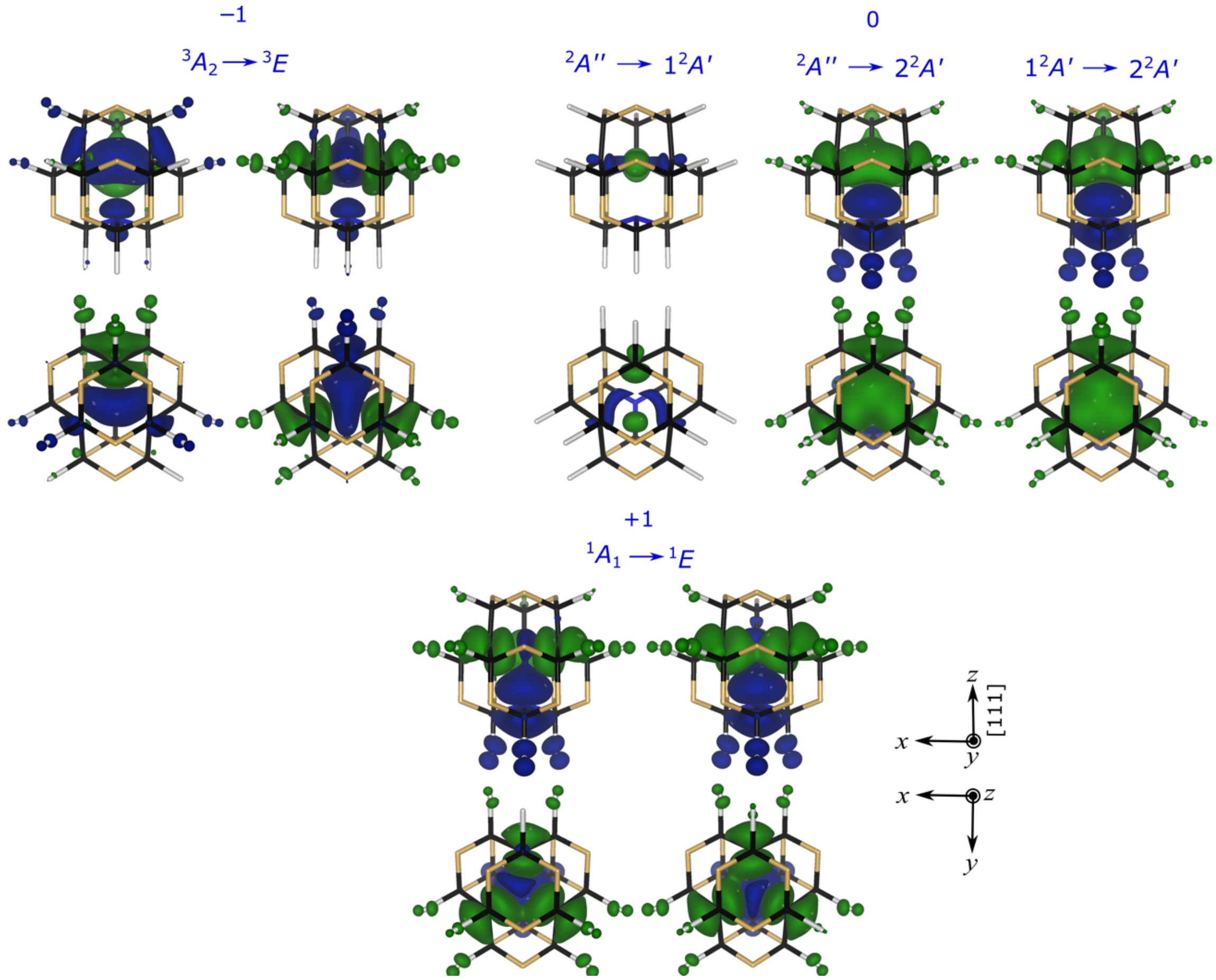

Fig. S13. **N_CV_C excited- and ground-state electron density difference ($\boldsymbol{\rho_j - \rho_i}$) plots**. State-specific densities from SA-CASSCF *n*-state-averaged density matrix (transition type as labelled) with *n* = 3 for charge and spin multiplicity (*q*,2*s*+1) = (–1,3), (0,2), and (+1,1), respectively. The plots are labelled by excitation type. Isosurface: green (gain)/blue (loss) = $\pm$ 0.002 e/bohr^3. The vectors are defined as: $x = [10\bar{1}], y = [\bar{1}2\bar{1}], z = [111]$ in the basis of the cubic lattice vectors of diamond.

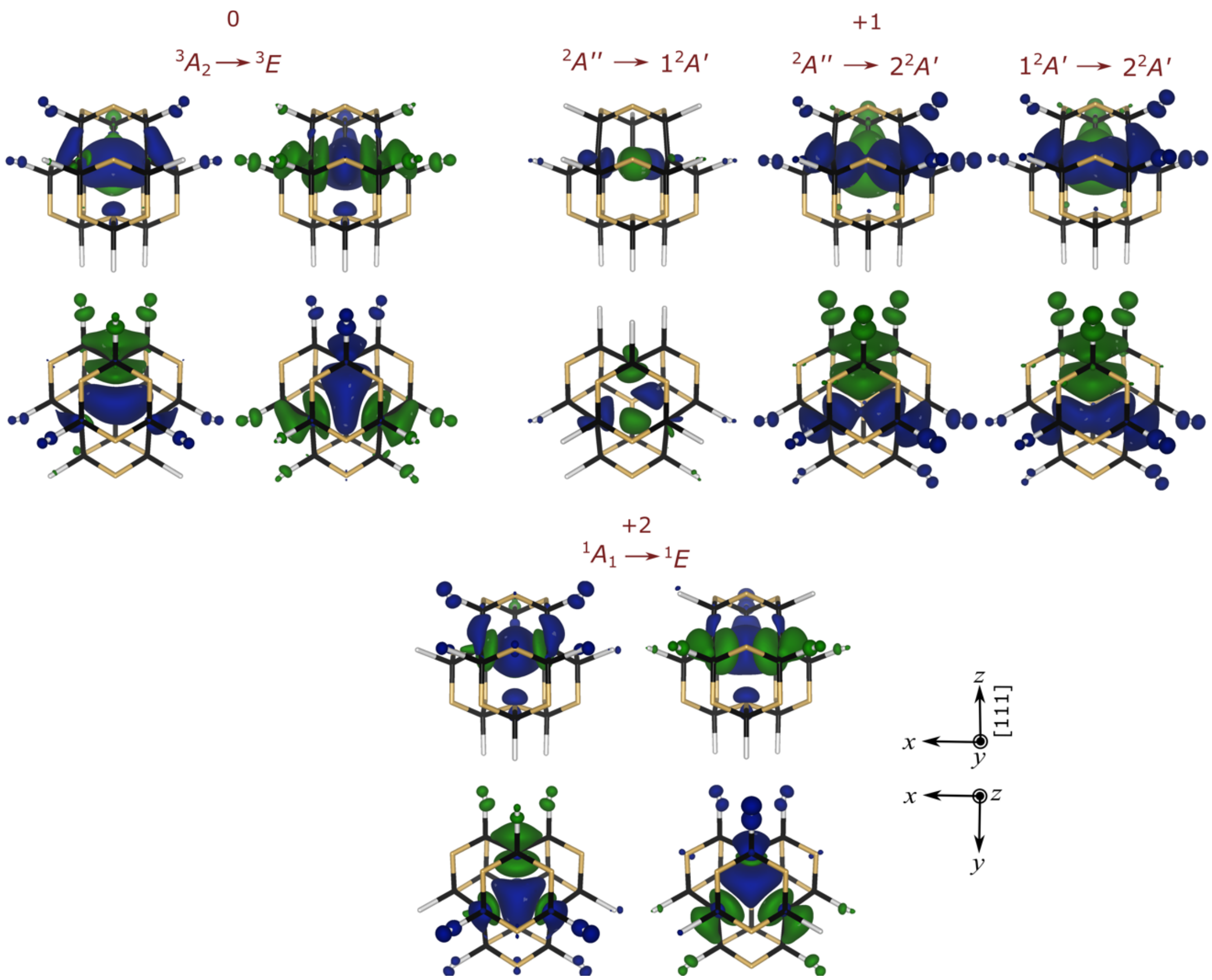

Fig. S14. O_CV_C excited- and ground-state electron density difference ($\rho_j - \rho_i$) plots. Densities from SA-CASSCF *n*-state-averaged density matrix (transition type as labelled) with *n* = 3, 3, and 3 for charge and spin multiplicity (q,2s+1) = (–1,3), (0,2), and (+1,1), respectively. The plots are labelled by excitation type. Isosurface: green (gain)/blue (loss) = $\pm$ 0.002 e/bohr^3. The vectors are defined as: $x = [10\bar{1}], y = [\bar{1}2\bar{1}], z = [111]$ in the basis of the cubic lattice vectors of diamond.

Table S1. Calculated[a] and experimental lattice constants for pure diamond.

Method	Cubic *a* (Å)	|Error|[b] (Å)
Experiment	3.56712 ± 0.00005[c]	--
DFT-r^2-SCAN-L	3.5677	0.0006
DFT-HSE06	3.5452	0.0225

[a]Using an eight-atom conventional cubic unit cell.
[b]Absolute error relative to experiment.
[c]X-ray diffraction at room temperature, ref. [18].

Table S2. DFT-r^2-SCAN-L-optimized lattice constants[a] for $C_{126}D$ supercells for all charge states at their respective ground-state spin.

D	Charge	|*a*|, |*b*|, |*c*| [Å]	α, β, γ [°]
B	-1	10.132, 10.132, 10.110	59.93, 59.93, 60.00
	0	10.114, 10.100, 10.092	59.95, 59.93, 59.96
	$+1$	10.078, 10.078, 10.068	59.97, 59.97, 60.00
C	-1	10.118, 10.118, 10.118	60.00, 60.00, 60.00
	0	10.101, 10.091, 10.091	60.07, 59.97, 59.97
N	-1	10.108, 10.108, 10.123	60.05, 60.05, 60.00
	0	10.092, 10.080, 10.098	60.07, 60.02, 59.96
	$+1$	10.053, 10.053, 10.076	60.07, 60.07, 60.00
O	0	10.091, 10.091, 10.098	60.02, 60.02, 60.00
	$+1$	10.074, 10.059, 10.076	60.05, 60.01, 59.95
	$+2$	10.035, 10.035, 10.053	60.06, 60.06, 60.00
pristine	0	10.091, 10.091, 10.091	60.00, 60.00, 60.00

[a]***a*** \|\| $[10\bar{1}]$; ***b*** \|\| $[01\bar{1}]$; ***c*** \|\| $[110]$ in the basis of the cubic unit lattice vectors.
Vectors were assigned so that the vector orthogonal to the plane
defined by vectors ***a*** and ***b*** plane is [111], i.e., $\boldsymbol{a} \times \boldsymbol{b}$ = [111].

Note S1. Ground-state spin splitting energies

Table S3 summarizes the ground-state spin-splitting energy between the ground spin state and a higher-energy spin state. The energies were both calculated from periodic 63-atom cubic supercells within DFT-HSE06 and $C_{15}DF_{12}O_{12}$ clusters/molecules carved from 127-atom primitive fcc supercells within emb-NEVPT2. The structures were optimized using DFT-r^2-SCAN-L (see Methods). The periodic DFT-HSE06 predictions likely exhibit strong defect-defect Coulomb interactions and therefore, likely non-converged with respect to cell size. However, these periodic models (with DFT-HSE06 level of theory) are adequate to determine the ground spin state of the defects with different charges prior to emb-NEVPT2 calculations. The qualitative trends found in the periodic DFT-HSE06 calculations from the 63-atom cubic supercells were corroborated by the emb-NEVPT2 calculations. The relative emb-NEVPT2 spin energies are consistently higher than that of the DFT-HSE06 predictions due to five key factors: (1) DFT tend to underestimate excited electronic states including higher spin states, (2) DFT cannot properly describe states exhibiting multiconfigurational character, (3) DFT converges to spin contaminated solutions, which artificially lower energies of higher-energy spin states by mixing-in some character of the lower-energy spin multiplicity, (4) the small 63-atom periodic supercell is not large enough to eliminate long-range defect-defect interactions that depends strongly on the distribution of the excess charge, which can change between different spin states, and (5) the supercells were optimized for each spin state for the smaller supercell but only for the ground spin state in the larger supercell. The first three factors likely primarily contribute to the quantitative discrepancy between DFT-HSE06 and emb-NEVPT2.

Table S3. Relative ground-state energies[a] of different spins within DFT-HSE06 (fixed s_z)[b] and emb-NEVPT2 (fixed s)[c]

Structure	Relative Energy [eV]			
	s_z; $s = 0$	$\frac{1}{2}$	1	$\frac{3}{2}$
		D = B		
$B_CV_C^{1-}$	0.33[d], 0.77		0.00	
$B_CV_C^{0}$		0.00		1.30, 1.65
$B_CV_C^{1+}$	0.00[e]		1.74, 2.29	
		D = C		
V_C^{1-}		0.32, 0.59		0.00
V_C^{0}	0.00[d]		0.12, 0.31	
		D = N		
$N_CV_C^{1-}$	0.31[d], 0.65		0.00	
$N_CV_C^{0}$		0.00		0.52, 0.75
$N_CV_C^{1+}$	0.00[e]		0.73, 1.11	
		D = O		
$O_CV_C^{0}$	0.33[d], 0.68		0.00	
$O_CV_C^{1+}$		0.00		0.92, 1.17
$O_CV_C^{2+}$	0.00[e]		1.33, 1.89	

[a]Ground-state spin multiplicity set as 0.[b]Fully optimized $C_{62}D$ cubic supercell for each charge and spins state within DFT-r^2-SCAN-L. In spin-unrestricted DFT, the wavefunctions are not guaranteed to be S^2 eigenfunctions but are S_z eigenfunctions.[c]Embedded $C_{15}DF_{12}O_{12}$ cluster from a fully optimized $C_{126}D$ cubic supercell within DFT-r^2-SCAN-L for the lowest-energy spin state of each charge state. Wavefunctions are expanded using configuration state functions and thus, are S^2 eigenfunctions. Ground-state-only means either a single-state (non-degenerate), two-state (doubly degenerate or nearly doubly degenerate), or three-state (triply or nearly triply degenerate) ground-state SA-CASSCF was performed for a given spin. [d]Open-shell spin singlet. [e]Closed-shell spin singlet.

Table S4. DFT-r^2-SCAN-L-optimized lattice constants[a] for $C_{62}D$ supercells for select charge states at their respective ground-state spin multiplicity and the C[3c]-C[3c] and C[3c]-D distances[b] at the defect site from both DFT-r^2-SCAN-L- and DFT-HSE06-optimized atomic structures.[c]

Defect	|**a**|, |**b**|, |**c**| [Å]	α, β, γ [°]	C[3c]-C[3c], C[3c]-D [Å]	
			**DFT-r^2-SCAN-L**	**DFT-HSE06**
$B_CV_C^{1-}$	7.179, 7.179, 7.179	89.74, 89.74, 90.26	2.728, 2.611	2.735, 2.620
V_C^{1-}	7.175, 7.175, 7.175	90.00, 90.00, 90.00	2.713, --	2.723, --
$N_CV_C^{1-}$	7.170, 7.170, 7.170	90.17, 90.17, 89.83	2.669, 2.742	2.678, 2.765
$O_CV_C^{0}$	7.140, 7.140, 7.140	90.09, 90.09, 89.91	2.653, 2.700	2.662, 2.738
pristine	7.135, 7.135, 7.135	90.00, 90.00, 90.00	--	--

[a]**a** || [100]; **b** || [010]; **c** || [001] in the basis of the cubic unit lattice vectors.
[b]Illustrated in top panels of Fig. 3 of the main text
[c]Using the DFT-r^2-SCAN-L-optimized lattice constant.

Note S2. Determination of the capped cluster symmetry group

Following the continuous symmetry measure method by Zabrodsky, Peleg, and Avnir,[19] given a symmetry group, G, the symmetry measure ($S'(G)$) is

$$S'(G) = \frac{1}{n_p}\sum_{i=1}^{n_p}\left\|P_i - \widehat{P_i}\right\|^2$$

where, n_p is the number of points in a structure and $\left\|P_i - \widehat{P_i}\right\|$ is the displacement of point P_i in the structure needed to reach the ideal point $\widehat{P_i}$ so that the structure belongs to the symmetry group G. The P_i vectors' origin are placed in the center of the structure and normalized by the maximum $\|P_i\|$. $\left\|P_i - \widehat{P_i}\right\|$ is a measure of the deviation of structure from a target shape. Therefore, the structure belongs to symmetry group G that minimizes $S'(G)$, with an ideal value of $S'(G) = 0$. It follows that each symmetry operation O_G belonging to group G will have $S'(O_G) = 0$ and max $\left\|P_i - \widehat{P_i}\right\| = 0$.

Table S5. Maximum normalized displacement $\|P_i - \widehat{P_i}\|$ for the six symmetry operations for the evaluation of the continuous symmetry measure for capped clusters [$C_{15}DF_{12}O_{12}$], D = B, N, and O exhibiting C_{3v} or C_s (distorted C_{3v}) point group symmetry

Structure & charge	max $\|P_i - \widehat{P_i}\|$ for symmetry operation[a]						$S'(C_{3v})$	Final assigned symmetry group
	**E** (x,y,z)	**C3** (-y,x-y,z)	**C3** (-x+y,-x,z)	**σ** (x-y,-y,z)	**σ** (y,x,z)	**σ** (-x,-x+y,z)		
D = B								
[$C_{15}BF_{12}O_{12}$]$^{1-}$	**0.0000**	**0.0003**	**0.0003**	**0.0003**	**0.0002**	**0.0002**	0.0000	C_{3v}
[$C_{15}BF_{12}O_{12}$]^{0}	**0.0000**	0.0300	0.0300	0.0264	**0.0005**	0.0262	0.0146	C_s
[$C_{15}BF_{12}O_{12}$]$^{1+}$	**0.0000**	**0.0000**	**0.0000**	**0.0000**	**0.0000**	**0.0000**	0.0000	C_{3v}
D = N								
[$C_{15}NF_{12}O_{12}$]$^{1-}$	**0.0000**	**0.0000**	**0.0000**	**0.0000**	**0.0000**	**0.0000**	0.0000	C_{3v}
[$C_{15}NF_{12}O_{12}$]^{0}	**0.0000**	0.0284	0.0284	0.0251	**0.0002**	0.0250	0.0131	C_s
[$C_{15}NF_{12}O_{12}$]$^{1+}$	**0.0000**	**0.0000**	**0.0000**	**0.0000**	**0.0000**	**0.0000**	0.0000	C_{3v}
D = O								
[$C_{15}OF_{12}O_{12}$]^{0}	**0.0000**	**0.0001**	**0.0001**	**0.0001**	**0.0000**	**0.0001**	0.0000	C_{3v}
[$C_{15}OF_{12}O_{12}$]$^{1+}$	**0.0000**	0.0350	0.0350	0.0307	**0.0003**	0.0305	0.0196	C_s
[$C_{15}OF_{12}O_{12}$]$^{2+}$	**0.0000**	**0.0000**	**0.0000**	**0.0000**	**0.0000**	**0.0000**	0.0000	C_{3v}

[a]maximum normalized displacement > 0.01 (1% max deviation) are greyed out. Such symmetry operations are removed, and the remaining symmetry operations dictate the final assigned symmetry group.

Table S6. Maximum normalized displacement $\|P_i - \widehat{P_i}\|$ for the 24 symmetry operations for the evaluation of the continuous symmetry measure for capped clusters [$C_{16}F_{12}O_{12}$] exhibiting T_d or D_{2d} (distorted T_d) point group symmetry[a]

Structure & charge	max $\|P_i - \widehat{P_i}\|$ for symmetry operation[a]						$S'(T_d)$	Final assigned symmetry group
	**E** (x,y,z)	**C3** (z,x,y)	**C3** (y,-z,-x)	**C3** (-z,-x,y)	**C3** (y,z,x)	**C3** (-y,z,-x)		
[$C_{16}F_{12}O_{12}$]$^{1-}$	**0.0000**	**0.0001**	**0.0001**	**0.0002**	**0.0001**	**0.0002**		
[$C_{16}F_{12}O_{12}$]^{0}	**0.0000**	0.0272	0.0272	0.0272	0.0272	0.0272		
	**C3** (-y,-z,x)	**C3** (z,-x,-y)	**C3** (-z,x,-y)	**C2** (-x,-y,z)	**C2** (x,-y,-z)	**C2** (-x,y,-z)		
[$C_{16}F_{12}O_{12}$]$^{1-}$	**0.0001**	**0.0001**	**0.0001**	**0.0001**	**0.0001**	**0.0001**		
[$C_{16}F_{12}O_{12}$]^{0}	0.0272	0.0272	0.0272	**0.0001**	**0.0001**	**0.0001**		
	**σ** (-z,y,-x)	**σ** (z,y,x)	**σ** (x,-z,-y)	**σ** (-x,z,y)	**σ** (y,x,z)	**σ** (-y,-x,z)		
[$C_{16}F_{12}O_{12}$]$^{1-}$	**0.0001**	**0.0001**	**0.0001**	**0.0001**	**0.0001**	**0.0001**		
[$C_{16}F_{12}O_{12}$]^{0}	0.0236	0.0237	0.0235	0.0235	**0.0002**	**0.0002**		
	**S4** (y,-x,-z)	**S4** (z,-y,-x)	**S4** (-x,z,-y)	**S4** (-x,-z,y)	**S4** (-y,x,-z)	**S4** (-z,-y,x)		
[$C_{16}F_{12}O_{12}$]$^{1-}$	**0.0001**	**0.0002**	**0.0002**	**0.0002**	**0.0001**	**0.0002**	0.0000	T_d
[$C_{16}F_{12}O_{12}$]^{0}	**0.0002**	0.0237	0.0235	0.0235	**0.0002**	0.0237	0.0190	D_{2d}

[a]maximum normalized displacement > 0.01 (1% max deviation) are greyed out. Such symmetry operations are removed, and the remaining symmetry operations dictate the final assigned symmetry group.

Table S7. Character tables for C_{3v}, C_s, T_d, and D_{2d} point group symmetries, as well as the corresponding irreps in the lower symmetry subgroup for C_{3v} and T_d (C_s and D_{2d}, respectively).[a]

$\boldsymbol{C_{3v}}$	E	$2C_3$	3σ	$\boldsymbol{C_{3v} \rightarrow C_s}$[b]			
A_1	1	1	1	A'			
A_2	1	1	-1	A''			
E	2	-1	0	$A' + A''$			
$\boldsymbol{C_s}$			σ				
A'			1				
A''			-1				
$\boldsymbol{T_d}$	E	$8C_3$	$3C_2$		$6S_4$	3σ	$\boldsymbol{T_d \rightarrow D_{2d}}$[b]
A_1	1	1	1		1	1	A_1
A_2	1	1	1		-1	-1	B_1
E	2	-1	2		0	0	$A_1 + B_1$
T_1	3	0	-1		1	-1	$A_2 + E$
T_2	3	0	-1		-1	1	$B_2 + E$
$\boldsymbol{D_{2d}}$	E		C_2	$2C'_2$	$2S_4$	2σ	
A_1	1		1	1	1	1	
A_2	1		1	-1	1	-1	
B_1	1		1	1	-1	-1	
B_2	1		1	-1	-1	1	
E	2		-2	0	0	0	

[a]Some of the symmetry operations are illustrated in **Fig. 3** in the main text.
[b]For *E* and *T* states, the states split into symmetry allowed states with lower degeneracies.

Table S8. Summary of relative energies and primary electronic configurations with $|c_i| > 0.40$ or until $\sum |c_i|^2 \geq 0.70$ for all states of B_CV_C and V_C in the basis of the primary valence SA-CASSCF natural orbitals (**Figs. S5** and **S6**).

Defect (AS)	State	Relative Energy[a] [eV]	Primary electronic configurations	c_i
$B_CV_C^{1-}$ (4e,7o)	$^3A_2$	0.000	$2a_1^2,2e^{1,1},3a_1^0$	0.94
	$1\ ^3E$	3.046<br>3.047	$2a_1^2,2e^{1,0},3a_1^1 \vert 2a_1^2,2e^{0,1},3a_1^1 \vert 2a_1^1,2e^{1,2},3a_1^0 \vert 2a_1^1,2e^{2,1},3a_1^0$<br>$2a_1^2,2e^{1,0},3a_1^1 \vert 2a_1^2,2e^{0,1},3a_1^1 \vert 2a_1^1,2e^{1,2},3a_1^0 \vert 2a_1^1,2e^{2,1},3a_1^0$	$0.49 \vert -0.46 \vert 0.38 \vert -0.35$<br>$0.46 \vert 0.48 \vert 0.35 \vert 0.38$
	$2\ ^3E$	3.795<br>3.803	$2a_1^1,2e^{2,1},3a_1^0 \vert 2a_1^2,2e^{0,1},3a_1^1$<br>$2a_1^1,2e^{1,2},3a_1^0 \vert 2a_1^2,2e^{1,0},3a_1^1$	$0.81 \vert -0.43$<br>$0.81 \vert -0.42$
	$1\ ^1E$	0.000<br>0.005	$2a_1^2,2e^{2,0},3a_1^0 \vert 2a_1^2,2e^{0,2},3a_1^0 \vert 2a_1^1,2e^{-1,2},3a_1^0 \vert 2a_1^2,2e^{1,0},3a_1^{-1}$<br>$2a_1^2,2e^{1,-1},3a_1^0 \vert 2a_1^2,2e^{0,1},3a_1^{-1}$	$0.56 \vert -0.55 \vert 0.35 \vert -0.33$<br>$0.78 \vert 0.33$
	$^1A_1$	1.386	$2a_1^2,2e^{2,0},3a_1^0 \vert 2a_1^2,2e^{0,2},3a_1^0 \vert 2a_1^0,2e^{2,2},3a_1^0$	$0.62 \vert 0.61 \vert -0.42$
	$2\ ^1E$	2.858<br>2.878	$2a_1^2,2e^{1,0},3a_1^{-1} \vert 2a_1^2,2e^{0,2},3a_1^0 \vert 2a_1^2,2e^{2,0},3a_1^0$<br>$2a_1^2,2e^{0,1},3a_1^{-1} \vert 2a_1^2,2e^{1,-1},3a_1^0$	$0.75 \vert -0.27 \vert 0.25$<br>$0.75 \vert -0.37$
$B_CV_C^0$ (3e,4o)	$^2A''$	0.000	$1a'^2,1a''^1,2a'^0, 3a'^0$	0.85
	$1\ ^2A'$	0.283	$1a'^2,1a''^0,2a'^1, 3a'^0$	0.83
	$2\ ^2A'$	3.223	$1a'^2,1a''^0,2a'^0, 3a'^1$	0.92
	$1\ ^4A''$	0.000	$1a'^1,1a''^1,2a'^1, 3a'^0$	1.00
	$^4A' -$ $2\ ^4A''$	3.809	$1a'^1,1a''^0,2a'^1, 3a'^1 \vert 1a'^1,1a''^1,2a'^0, 3a'^1$	$0.71 \vert -0.71$
	$^4A' +$ $2\ ^4A''$	4.060	$1a'^1,1a''^0,2a'^1, 3a'^1 \vert 1a'^1,1a''^1,2a'^0, 3a'^1$	$0.71 \vert 0.71$
$B_CV_C^{1+}$ (2e,3o)	$^1A_1$	0.000	$2a_1^2,2e^{0,0}$	0.97
	^1E	3.240<br>3.250	$2a_1^1,2e^{0,-1} \vert 2a_1^0,2e^{1,-1}$<br>$2a_1^1,2e^{-1,0} \vert 2a_1^0,2e^{0,2} \vert 2a_1^0,2e^{2,0}$	$0.76 \vert -0.60$<br>$0.76 \vert -0.42 \vert 0.42$
	^3E	0.000<br>0.031	$2a_1^1,2e^{1,0} \vert 2a_1^1,2e^{0,1}$<br>$2a_1^1,2e^{1,0} \vert 2a_1^1,2e^{0,1}$	$0.67 \vert 0.69$<br>$0.73 \vert -0.68$
	$^3A_2$	2.670	$2a_1^0,2e^{1,1}$	0.95
V_C^{1-} (5e,8o)	$^4A_2$	0.000	$1a_1^2,1t_2^{1,1,1}$	0.98
	$^4T_1$	3.368<br>3.371<br>3.400	$1a_1^1,1t_2^{1,1,2}$<br>$1a_1^1,1t_2^{1,2,1}$<br>$1a_1^1,1t_2^{2,1,1}$	0.96<br>0.95<br>0.95
	$^2T_1$	0.000<br>0.002<br>0.010	$1a_1^2,1t_2^{1,2,0} \vert 1a_1^2,1t_2^{1,0,2} \vert 1a_1^1,1t_2^{-1,1,2}$<br>$1a_1^2,1t_2^{1,1,-1} \vert 1a_1^2,1t_2^{0,2,1} \vert 1a_1^2,1t_2^{2,0,1}$<br>$1a_1^2,1t_2^{1,1,-1} \vert 1a_1^2,1t_2^{0,2,1} \vert 1a_1^2,1t_2^{2,0,1} \vert 1a_1^2,1t_2^{1,-1,1}$	$0.58 \vert -0.58 \vert -0.27$<br>$0.72 \vert 0.37 \vert -0.38$<br>$0.34 \vert -0.41 \vert 0.41 \vert 0.60$
	^2E	1.432<br>1.432	$1a_1^2,1t_2^{2,1,0} \vert 1a_1^1,1t_2^{2,0,2} \vert 1a_1^2,1t_2^{0,1,2} \vert 1a_1^1,1t_2^{1,-1,2} \vert 1a_1^1,1t_2^{2,2,0}$<br>$1a_1^1,1t_2^{1,2,-1} \vert 1a_1^2,1t_2^{1,-1,1} \vert 1a_1^1,1t_2^{-1,2,1} \vert 1a_1^2,1t_2^{1,1,-1}$	$0.47 \vert 0.46 \vert -0.44 \vert 0.26 \vert -0.25$<br>$0.55 \vert -0.48 \vert -0.32 \vert 0.30$
	$^2T_2$	1.770<br>1.772<br>1.779	$1a_1^2,1t_2^{0,2,1} \vert 1a_1^2,1t_2^{2,0,1} \vert 1a_1^0,1t_2^{2,2,1}$<br>$1a_1^2,1t_2^{0,1,2} \vert 1a_1^2,1t_2^{2,1,0} \vert 1a_1^0,1t_2^{2,1,2}$<br>$1a_1^2,1t_2^{1,0,2} \vert 1a_1^2,1t_2^{1,2,0} \vert 1a_1^0,1t_2^{1,2,2}$	$0.53 \vert 0.52 \vert -0.38$<br>$0.53 \vert 0.51 \vert -0.37$<br>$0.55 \vert 0.54 \vert -0.39$
V_C^0 (4e,8o)	$^1B_1$	0.000	$1a_1^2,2e^{1,-1},1b_2^0$	0.84
	$^1A_1$	0.215	$1a_1^2,2e^{0,0},1b_2^2 \vert 1a_1^2,2e^{0,2},1b_2^0 \vert 1a_1^2,2e^{2,0},1b_2^0$	$0.64 \vert -0.35 \vert -0.34$
	$^1B_2$	1.726	$1a_1^2,2e^{2,0},1b_2^0 \vert 1a_1^2,2e^{0,2},1b_2^0$	$0.69 \vert -0.61$
	^1E	1.917<br>1.950	$1a_1^2,2e^{1,0},1b_2^{-1}$<br>$1a_1^2,2e^{0,1},1b_2^{-1}$	0.89<br>0.90
	$1\ ^3A_2$	0.000	$1a_1^2,2e^{1,1},1b_2^0 \vert 1a_1^1,2e^{1,-1},1b_2^1$	$0.77 \vert 0.50$
	$1\ ^3E$	0.146<br>0.166	$1a_1^2,2e^{1,0},1b_2^1 \vert 1a_1^1,2e^{1,2},1b_2^0 \vert 1a_1^1,2e^{1,0},1b_2^2$<br>$1a_1^2,2e^{0,1},1b_2^1 \vert 1a_1^1,2e^{2,1},1b_2^0 \vert 1a_1^1,2e^{0,1},1b_2^2$	$0.75 \vert -0.43 \vert 0.41$<br>$0.76 \vert 0.42 \vert -0.39$
	$2\ ^3E$	2.812<br>2.817	$1a_1^2,2e^{1,0},1b_2^1 \vert 1a_1^1,2e^{1,2},1b_2^0 \vert 1a_1^1,2e^{1,0},1b_2^2$<br>$1a_1^2,2e^{1,1},1b_2^0 \vert 1a_1^1,2e^{1,-1},1b_2^1$	$0.63 \vert 0.54 \vert -0.47$<br>$-0.59 \vert 0.62$
	$2\ ^3A_2$	2.834	$1a_1^2,2e^{0,1},1b_2^1 \vert 1a_1^1,2e^{2,1},1b_2^0 \vert 1a_1^1,2e^{0,1},1b_2^2$	$0.61 \vert -0.54 \vert 0.44$

[a]Relative to the lowest-energy state for a given spin multiplicity

Table S9. Summary of relative energies and primary electronic configurations with $|c_i|$ > 0.40 or until $\sum |c_i|^2 \geq 0.70$ for all states of N_CV_C and O_CV_C in the basis of the primary valence SA-CASSCF natural orbitals (**Figs. S7** and **S8**).

<table>
<tr><th>Defect (AS)</th><th>State</th><th>Relative Energy[a] [eV]</th><th>Primary electronic configurations</th><th>c_i</th></tr>
<tr><td rowspan="4">$N_CV_C^{1-}$ (6e,8o)</td><td>$^3A_2$</td><td>0.000</td><td>$2a_1^2,3a_1^2,2e^{1,1}$</td><td>0.98</td></tr>
<tr><td>^3E</td><td>2.251
2.273</td><td>$2a_1^2,3a_1^1,2e^{1,2}$
$2a_1^2,3a_1^1,2e^{2,1}$</td><td>0.94
0.94</td></tr>
<tr><td>^1E</td><td>0.000
0.013</td><td>$2a_1^2,3a_1^2,2e^{2,0}$ | $2a_1^2,3a_1^2,2e^{0,2}$ | $2a_1^2,3a_1^1,2e^{-1,2}$
$2a_1^2,3a_1^2,2e^{1,-1}$ | $2a_1^2,3a_1^1,2e^{2,-1}$</td><td>0.62|-0.61|-0.43
0.87|0.43</td></tr>
<tr><td>$^1A_1$</td><td>1.272</td><td>$2a_1^2,3a_1^2,2e^{2,0}$ | $2a_1^2,3a_1^2,2e^{0,2}$ | $2a_1^2,3a_1^0,2e^{2,2}$</td><td>0.57|0.58|-0.53</td></tr>
<tr><td rowspan="5">$N_CV_C^0$ (5e,6o)</td><td>$1^2A''$</td><td>0.000</td><td>$1a'^2,2a'^2,1a''^1,3a'^0$ | $1a'^2,2a'^0,1a''^1,3a'^2$ | $1a'^2,2a'^1,1a''^{-1},3a'^1$</td><td>0.78|-0.32|0.31</td></tr>
<tr><td>$^2A'$</td><td>0.161</td><td>$1a'^2,2a'^2,1a''^0,3a'^1$ | $1a'^2,2a'^1,1a''^0,3a'^2$ | $1a'^2,2a'^1,1a''^2,3a'^0$</td><td>0.70|0.40|-0.39</td></tr>
<tr><td>$2\,^2A''$</td><td>2.737</td><td>$1a'^2,2a'^1,1a''^{-1},3a'^1$ | $1a'^1,2a'^2,1a''^{-1},3a'^1$ | $1a'^2,2a'^1,1a''^1,3a'^{-1}$</td><td>0.70|0.47|0.38</td></tr>
<tr><td>$1\,^4A''$</td><td>0.000</td><td>$1a'^2,2a'^1,1a''^1,3a'^1$ | $1a'^1,2a'^2,1a''^1,3a'^1$</td><td>0.89|-0.43</td></tr>
<tr><td>$2\,^4A''$</td><td>3.261</td><td>$1a'^2,2a'^1,1a''^1,3a'^1$ | $1a'^1,2a'^2,1a''^1,3a'^1$</td><td>0.42|0.88</td></tr>
<tr><td rowspan="5">$N_CV_C^{1+}$ (4e,5o)</td><td>$^1A_1$</td><td>0.000</td><td>$2a_1^2,3a_1^2,2e^{0,0}$</td><td>0.93</td></tr>
<tr><td>^1E</td><td>2.063
2.057</td><td>$2a_1^2,3a_1^1,2e^{0,-1}$ | $2a_1^2,3a_1^0,2e^{1,-1}$
$2a_1^2,3a_1^1,2e^{-1,0}$ | $2a_1^2,3a_1^0,2e^{0,2}$ | $2a_1^2,3a_1^0,2e^{2,0}$</td><td>0.70|0.44
0.70|0.32|-0.30</td></tr>
<tr><td>$1\,^3E$</td><td>0.000
0.047</td><td>$2a_1^2,3a_1^1,2e^{1,0}$ | $2a_1^1,3a_1^2,2e^{1,0}$
$2a_1^2,3a_1^1,2e^{0,1}$ | $2a_1^1,3a_1^2,2e^{0,1}$</td><td>0.76|0.43
0.76|0.44</td></tr>
<tr><td>$2\,^3E$</td><td>1.266
1.278</td><td>$2a_1^2,3a_1^1,2e^{1,0}$ | $2a_1^1,3a_1^2,2e^{1,0}$
$2a_1^2,3a_1^1,2e^{0,1}$ | $2a_1^1,3a_1^2,2e^{0,1}$</td><td>-0.62|0.65
-0.62|0.64</td></tr>
<tr><td>$^3A_2$</td><td>1.408</td><td>$2a_1^2,3a_1^0,2e^{1,1}$ | $2a_1^1,3a_1^1,2e^{-1,1}$ | $2a_1^1,3a_1^{-1},2e^{1,1}$</td><td>0.72|-0.41|-0.36</td></tr>
<tr><td rowspan="4">$O_CV_C^0$ (4e,6o)</td><td>$^3A_2$</td><td>0.000</td><td>$3a_1^2,2e^{1,1}$</td><td>0.98</td></tr>
<tr><td>^3E</td><td>2.638
2.653</td><td>$3a_1^1,2e^{1,2}$
$3a_1^1,2e^{2,1}$</td><td>0.90
0.90</td></tr>
<tr><td>^1E</td><td>0.000
0.010</td><td>$3a_1^2,2e^{2,0}$ | $3a_1^2,2e^{0,2}$ | $3a_1^1,2e^{-1,2}$
$3a_1^2,2e^{1,-1}$ | $3a_1^1,2e^{2,-1}$</td><td>0.63|-0.62|0.42
0.88|-0.42</td></tr>
<tr><td>$^1A_1$</td><td>1.285</td><td>$3a_1^2,2e^{2,0}$ | $3a_1^2,2e^{0,2}$ | $3a_1^0,2e^{2,2}$</td><td>0.58|0.59|-0.50</td></tr>
<tr><td rowspan="4">$O_CV_C^{1+}$ (3e,4o)</td><td>$^2A''$</td><td>0.000</td><td>$2a'^2,1a''^1,3a'^0$ | $2a'^1,1a''^{-1},3a'^1$</td><td>0.77|-0.44</td></tr>
<tr><td>$1\,^2A'$</td><td>0.146</td><td>$2a'^2,1a''^0,3a'^1$ | $2a'^1,1a''^0,3a'^2$ | $2a'^1,1a''^2,3a'^0$</td><td>0.64|0.53|-0.44</td></tr>
<tr><td>$2\,^2A'$</td><td>3.410</td><td>$2a'^2,1a''^0,3a'^1$ | $2a'^1,1a''^0,3a'^2$ | $2a'^1,1a''^2,3a'^0$</td><td>0.74|-0.55|0.32</td></tr>
<tr><td>$^4A''$</td><td>0.000</td><td>$2a'^1,1a''^1,3a'^1$</td><td>1.00</td></tr>
<tr><td rowspan="4">$O_CV_C^{2+}$ (2e,3o)</td><td>$^1A_1$</td><td>0.000</td><td>$3a_1^2,2e^{0,0}$</td><td>0.95</td></tr>
<tr><td>^1E</td><td>2.819
2.839</td><td>$3a_1^1,2e^{0,-1}$ | $3a_1^0,2e^{1,-1}$
$3a_1^1,2e^{-1,0}$ | $3a_1^0,2e^{0,2}$ | $3a_1^0,2e^{2,0}$</td><td>0.75|-0.64
0.75|-0.45|0.45</td></tr>
<tr><td>^3E</td><td>0.000
0.035</td><td>$3a_1^1,2e^{1,0}$ | $3a_1^1,2e^{0,1}$ | $3a_1^0,2e^{1,1}$
$3a_1^1,2e^{1,0}$ | $3a_1^1,2e^{0,1}$ | $3a_1^0,2e^{1,1}$</td><td>0.82|0.43|-0.37
-0.56|0.65|0.50</td></tr>
<tr><td>$^3A_2$</td><td>2.296</td><td>$3a_1^1,2e^{0,1}$ | $3a_1^0,2e^{1,1}$</td><td>0.63|0.78</td></tr>
</table>

[a]Relative to the lowest-energy state for a given spin multiplicity

Table S10. Symmetry product table for C_{3v} and C_s point group symmetries.

C_{3v}	A_1	A_2	E
A_1	A_1	A_2	E
A_2	A_2	A_1	E
E	E	E	A_1+A_2+E
C_s	A'	A''	
A'	A'	A''	
A''	A''	A'	

Table S11. Allowed states for V_C with -1 and 0 charges and T_d point group symmetry.[a]

Charge	No. valence electrons	Valence electronic configuration	Allowed states
-1	5	$a_1^0t_2^5$	$^2T_2$
		$a_1^1t_2^4$	$^2A_1 + {}^2E + {}^4T_1 + {}^2T_1 + {}^2T_2$
		$a_1^2t_2^3$	$^4A_2 + {}^2E + {}^2T_1 + {}^2T_2$
0	4	$a_1^2t_2^2$ & $a_1^0t_2^4$	$^1A_1 + {}^1E + {}^3T_1 + {}^1T_2$
		$a_1^1t_2^3$	$^5A_2 + {}^3A_2 + {}^3E + {}^1E + {}^3T_1 + {}^1T_1 + {}^3T_2 + {}^1T_2$

[a]Data taken from ref. [20].

Table S12. Transition dipole moment (μ_t) vector components and magnitude, emission lifetime (τ), and oscillator strength (f) for select electronic excitations (as labelled) of the ground-state spin multiplicity of all defects and their charges.

Defect	Excitation	Transition dipole[a] [D]				τ [ns]	f[b]
		$\mu_{t,x}$	$\mu_{t,y}$	$\mu_{t,z}$	$\lvert\boldsymbol{\mu}_t\rvert$		
$B_CV_C^{1-}$	$^3A_2$ → 1 ^3E (1)	**-0.9261**	**-1.2477**	0.0077	1.5539	36	0.028
	$^3A_2$ → 1 ^3E (2)	**-1.2256**	**0.9442**	-0.0086	1.5472	36	0.028
	$^3A_2$ → 2 ^3E (1)	-0.1931	**3.8938**	-0.0657	3.8991	2.9	0.22
	$^3A_2$ → 2 ^3E (2)	**-3.8872**	-0.1992	0.0041	3.8923	2.9	0.22
$B_CV_C^{0}$	$^2A''$ → 1 $^2A'$	**-0.4668**	-0.0060	0.0006	0.4669	-	-
	$^2A''$ → 2 $^2A'$	**0.5837**	-0.0078	0.0002	0.5837	215	0.0042
	1 $^2A'$ → 2 $^2A'$	0.0034	**0.7773**	-0.0950	0.7831	159	0.0068
$B_CV_C^{1+}$	$^1A_1$ → ^1E (1)	**-5.4549**	**1.4833**	-0.0056	5.6529	2.3	0.38
	$^1A_1$ → ^1E (2)	**-1.4873**	**-5.4386**	0.0091	5.6383	2.3	0.39
V_C^{1-}	$^4A_2$ → $^4T_1$ (1)	0.0043	**1.1416**	**-4.3637**	4.5106	3.2	0.26
	$^4A_2$ → $^4T_1$ (2)	-0.0494	**4.3422**	**1.1384**	4.4892	3.2	0.26
	$^4A_2$ → $^4T_1$ (3)	**-4.4764**	-0.0527	-0.0213	4.4768	3.1	0.26
V_C^{0}	$^1B_1$ → $^1A_1$	-0.0051	-0.0004	0.0006	0.0052	-	-
	$^1B_1$ → $^1B_2$	**0.2360**	0.0037	0.0036	0.2361	8.8×10^3	0.00036
	$^1B_1$ → ^1E (1)	**-1.1868**	0.0003	-0.0056	1.1868	254	0.010
	$^1B_1$ → ^1E (2)	0.0076	**0.6729**	**-0.8708**	1.1005	280	0.0090
	$^1A_1$ → $^1B_2$	-0.0047	**-1.1303**	**-0.6510**	1.3044	431	0.0097
	$^1A_1$ → ^1E (1)	-0.0071	-0.0234	**-0.4682**	0.4688	2.3×10^3	0.0014
	$^1A_1$ → ^1E (2)	**-0.5956**	0.0103	0.0063	0.5957	1.4×10^3	0.0023
$N_CV_C^{1-}$	$^3A_2$ → ^3E (1)	0.1192	**4.7904**	-0.0040	4.7919	9.6	0.20
	$^3A_2$ → ^3E (2)	**-4.7560**	0.1128	-0.0017	4.7573	9.4	0.20
$N_CV_C^{0}$	1 $^2A''$ → $^2A'$	**-0.1787**	-0.0015	0.0000	0.1787	-	-
	1 $^2A''$ → 2 $^2A''$	-0.0036	**-0.3013**	**-0.1338**	0.3297	1.1×10^3	0.0011
	$^2A'$ → 2 $^2A''$	**0.3315**	-0.0004	-0.0012	0.3315	1.3×10^3	0.0011
$N_CV_C^{1+}$	$^1A_1$ → ^1E (1)	**1.8359**	**-0.5619**	**0.2011**	1.9305	77	0.029
	$^1A_1$ → ^1E (2)	**0.5955**	**1.7196**	**-0.5712**	1.9073	79	0.028
$O_CV_C^{0}$	$^3A_2$ → ^3E (1)	-0.1613	**-4.9282**	0.0028	4.9309	5.6	0.24
	$^3A_2$ → ^3E (2)	**-4.9035**	0.1536	-0.0029	4.9059	5.5	0.24
$O_CV_C^{1+}$	$^2A''$ → 1 $^2A'$	**0.3274**	-0.0669	0.0011	0.3341	-	-
	$^2A''$ → 2 $^2A'$	**-1.6579**	**0.7709**	-0.0126	1.8284	18	0.043
	1 $^2A'$ → 2 $^2A'$	**0.2450**	**-2.2057**	0.0365	2.2196	14	0.061
$O_CV_C^{2+}$	$^1A_1$ → ^1E (1)	**5.4996**	-0.2678	0.0004	5.5061	3.7	0.32
	$^1A_1$ → ^1E (2)	-0.2705	**-5.4636**	0.0013	5.4702	3.6	0.32

[a]Dominant μ_t component(s) are in **boldface**. The vectors are defined as: $x = [10\bar{1}], y = [\bar{1}2\bar{1}], z = [111]$ in the basis of the cubic lattice vectors of diamond. [b]For degenerate excited states, to obtain the effective f, one should take the sum of the individual fs of the states that are degenerate (**Eq. S8**).

Supplementary References

(1) Kresse, G.; Fürthmuller, J. Efficient iterative schemes for ab initio total-energy calculations using a plane-wave basis set. *Physical Review B* **1996**, *54* (16), 11169-11186.
(2) Kresse, G.; Joubert, D. From ultrasoft pseudopotentials to the projector augmented-wave method. *Physical Review B* **1999**, *59* (3), 1758-1775.
(3) Blöchl, P. E. Projector augmented-wave method. *Phys. Rev. B* **1994**, *50* (24), 17953-17979.
(4) Martirez, J. M. P. Optical properties of a diamond NV color center from capped embedded multiconfigurational correlated wavefunction theory. *npj Computational Materials* **2026**, *12*, 113.
(5) Martirez, J. M. P.; Carter, E. A. Projector-Free Capped-Fragment Scheme within Density Functional Embedding Theory for Covalent and Ionic Compounds. *Journal of Chemical Theory and Computation* **2021**, *17* (7), 4105–4121.
(6) Chung, L. W.; Sameera, W. M. C.; Ramozzi, R.; Page, A. J.; Hatanaka, M.; Petrova, G. P.; Harris, T. V.; Li, X.; Ke, Z. F.; Liu, F. Y.; Li, H. B.; Ding, L. N.; Morokuma, K. The ONIOM Method and Its Applications. *Chemical Reviews* **2015**, *115* (12), 5678-5796.
(7) Wu, Q.; Yang, W. A Direct Optimization Method for Calculating Density Functionals and Exchange–Correlation Potentials from Electron Densities. *Journal of Chemical Physics* **2003**, *118*, 2498-2509.
(8) Yu, K.; Libisch, F.; Carter, E. A. Implementation of density functional embedding theory within the projector-augmented-wave method and applications to semiconductor defect states. *Journal of Chemical Physics* **2015**, *143* (10), 102806.
(9) Yu, K.; Libisch, F.; Schlipf, M.; Boyn, J.-N.; Martirez, J. M. P.; Carter, E. A. *VASPEmbedding*. 2024. https://github.com/EACcodes/VASPEmbedding (accessed 2024 October).
(10) Werner, H.-J.; Knowles, P. J.; Knizia, G.; Manby, F. R.; Schütz, M. Molpro: a general-purpose quantum chemistry program package. *WIREs Comput. Mol. Sci.* **2012**, *2* (2), 242-253.
(11) Werner, H.-J.; Knowles, P. J.; Manby, F. R.; Black, J. A.; Doll, K.; Heßelmann, A.; Kats, D.; Köhn, A.; Korona, T.; Kreplin, D. A.; Ma, Q.; Miller, T. F., III; Mitrushchenkov, A.; Peterson, K. A.; Polyak, I.; Rauhut, G.; Sibaev, M. The Molpro quantum chemistry package. *The Journal of Chemical Physics* **2020**, *152* (14), 144107.
(12) Dunning, T. H. Gaussian-basis sets for use in correlated molecular calculations. 1. The atoms boron through neon and hydrogen. *Journal of Chemical Physics* **1989**, *90* (2), 1007-1023.
(13) Krauter, C. M.; Zhang, X.; Carter, E. A. *Embedding Integral Generator*. https://github.com/EACcodes/EmbeddingIntegralGenerator (accessed 2024 April).
(14) Angeli, C.; Cimiraglia, R.; Evangelisti, S.; Leininger, T.; Malrieu, J. P. Introduction of n-electron valence states for multireference perturbation theory. *J. Chem. Phys.* **2001**, *114* (23), 10252-10264.
(15) Sarkar, R.; Loos, P.-F.; Boggio-Pasqua, M.; Jacquemin, D. Assessing the Performances of CASPT2 and NEVPT2 for Vertical Excitation Energies. *Journal of Chemical Theory and Computation* **2022**, *18* (4), 2418-2436.
(16) Stoneham, A. M. *Theory of Defects in Solids: Electronic Structure of Defects in Insulators and Semiconductors*; Oxford University Press, 2001.
(17) Thomas, M.; Tropf, W. *Optical properties of diamond*; SPIE, 1994.
(18) Yamanaka, T.; Morimoto, S. Isotope effect on anharmonic thermal atomic vibration and [kappa] refinement of 12C and 3C diamond. *Acta Crystallographica Section B* **1996**, *52* (2), 232-238.
(19) Zabrodsky, H.; Peleg, S.; Avnir, D. Continuous symmetry measures. 2. Symmetry groups and the tetrahedron. *Journal of the American Chemical Society* **1993**, *115* (18), 8278-8289.
(20) Coulson, C. A.; Kearsley, M. J. Colour centres in irradiated diamonds. I. *Proceedings of the Royal Society of London. A. Mathematical and Physical Sciences* **1957**, *241* (1227), 433-454.(acccessed 1/4/2026).
